# Physicochemical investigation of a novel curcumin diethyl γ-aminobutyrate, a carbamate ester prodrug of curcumin with enhanced anti-neuroinflammatory activity

**DOI:** 10.1371/journal.pone.0265689

**Published:** 2022-03-18

**Authors:** Ponsiree Jithavech, Piyapan Suwattananuruk, Hasriadi, Chawanphat Muangnoi, Worathat Thitikornpong, Pasarapa Towiwat, Opa Vajragupta, Pornchai Rojsitthisak

**Affiliations:** 1 Center of Excellence in Natural Products for Ageing and Chronic Diseases, Chulalongkorn University, Bangkok, Thailand; 2 Department of Food and Pharmaceutical Chemistry, Faculty of Pharmaceutical Sciences, Chulalongkorn University, Bangkok, Thailand; 3 Pharmaceutical Sciences and Technology Program, Faculty of Pharmaceutical Sciences, Chulalongkorn University, Bangkok, Thailand; 4 Cell and Animal Model Unit, Institute of Nutrition, Mahidol University, Nakhon Pathom, Thailand; 5 Department of Pharmacology and Physiology, Faculty of Pharmaceutical Sciences, Chulalongkorn University, Bangkok, Thailand; 6 Research Affairs, Faculty of Pharmaceutical Sciences, Chulalongkorn University, Bangkok, Thailand; Tribhuvan University, NEPAL

## Abstract

Curcumin is a polyphenol compound that alleviates several neuroinflammation-related diseases including Alzheimer’s disease, Parkinson’s disease, multiple sclerosis, epilepsy and cerebral injury. However, the therapeutic efficacy of curcumin is limited by its poor physicochemical properties. The present study aimed to develop a new carrier-linked curcumin prodrug, curcumin diethyl γ-aminobutyrate (CUR-2GE), with improved physicochemical and anti-neuroinflammatory properties. CUR-2GE was designed and synthesized by conjugating curcumin with gamma-aminobutyric acid ethyl ester (GE) via a carbamate linkage. The carbamate linkage was selected to increase stability at acidic pH while GE served as a promoiety for lipophilic enhancement. The synthesized CUR-2GE was investigated for solubility, partition coefficient, stability, and bioconversion. The solubility of CUR-2GE was less than 0.05 μg/mL similar to that of curcumin, while the lipophilicity with log P of 3.57 was significantly increased. CUR-2GE was resistant to chemical hydrolysis at acidic pH (pH 1.2 and 4.5) as anticipated but rapidly hydrolyzed at pH 6.8 and 7.4. The incomplete hydrolysis of CUR-2GE was observed in simulated gastrointestinal fluids which liberated the intermediate curcumin monoethyl γ-aminobutyric acid (CUR-1GE) and the parent curcumin. In plasma, CUR-2GE was sequentially converted to CUR-1GE and curcumin within 1 h. In lipopolysaccharide (LPS)-stimulated BV-2 microglial cells, CUR-2GE effectively attenuated the pro-inflammatory mediators by decreasing the secretion of nitric oxide and cytokines (TNF-α and IL-6) to a greater extent than curcumin due to an increase in cellular uptake. Altogether, the newly developed acid-stable CUR-2GE prodrug is a potential pre-clinical and clinical candidate for further evaluation on neuroprotective and anti-neuroinflammatory effects.

## Introduction

Neuroinflammation is a major contributing factor to the pathophysiology of several CNS diseases, including Alzheimer’s disease, Parkinson’s disease, multiple sclerosis, epilepsy and cerebral injury [1, 2]. Microglia, a resident immune cell in CNS, plays an essential role in maintaining the homeostasis of the CNS and is involved in the progression of neuroinflammation-associated diseases [3, 4]. In this pathophysiological condition, microglia are activated, leading to the magnificent release of several pro-inflammatory mediators, including cytokines, chemokines, growth factors, NO, and PGE-2 [5, 6]. Thus, abrogating pro-inflammatory mediators released by microglia is a possible option to improve neuroinflammation-associated diseases.

**Fig 1:**
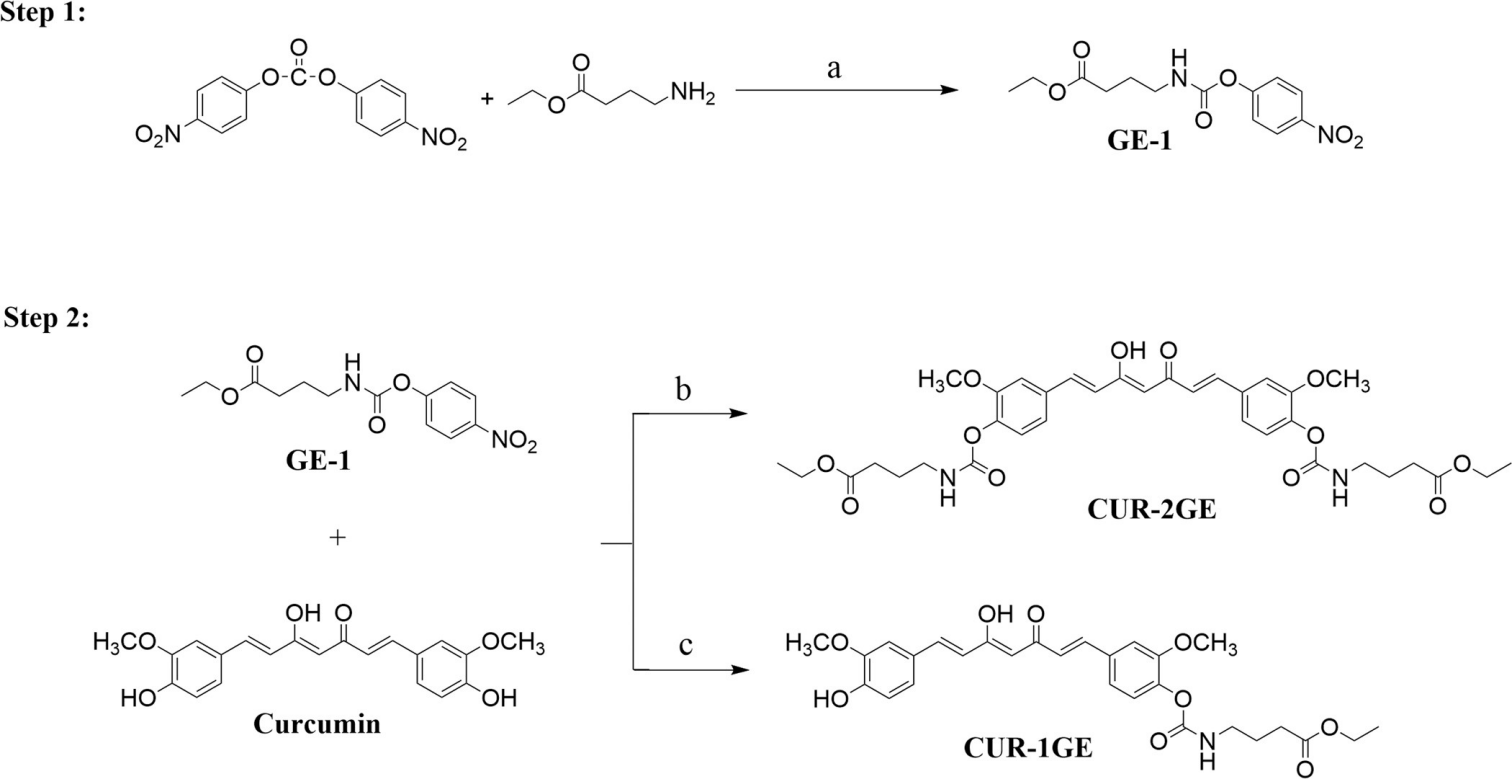
Synthesis of CUR-2GE and CUR-1GE. Reagents and condition (a) DMAP/ACN 50°C 3 h (b) DMAP/ACN 50°C 24 h with GE-1: curcumin at molar ratio 4.5: 1 (c) DMAP/ACN 50°C 24 h with GE-1: curcumin at molar ratio 1: 2.5.

Curcumin (Fig 1) has been shown to alleviate several neuroinflammation-related diseases [7–9]. At the cellular level, curcumin modulated microglia by reducing pro-inflammatory mediators and increasing endogenous anti-inflammatory mediators [10, 11]. The extremely low oral bioavailability of curcumin due to its chemical and metabolic instability is the primary limiting factor for oral nutraceutical and pharmaceutical development. Several approaches have been employed and applied to overcome the above disadvantages of curcumin such as nanoparticles [12–15], polymer conjugates [16], and dicarboxylate prodrugs [17–19].

Prodrugs are pharmacologically inactive substances that undergo metabolic or chemical conversion to active parent molecules [20, 21]. In drug development, prodrug performance is commonly optimized by modulating physicochemical and biopharmaceutical properties through the addition of a promoiety to the parent drug via an appropriate linkage. The type of linkage is mainly associated with the persistence of the prodrug whereas the promoiety generally influences solubility or lipophilicity of the parent drug [22, 23]. Several curcumin prodrugs with an ester linkage showed the improvement of stability, permeability and efficacy such as dicarboxylate conjugates (curcumin diethyl disuccinate, curcumin diethyl diglutarate and curcumin diglutaric acid) and polymer conjugates (curcumin-monomethoxy polyethylene glycol) [7, 16–19, 24].

In addition to the extensively developed ester prodrugs of various bioactive molecules, carbamate prodrugs have gained much interest in drug design and discovery. It is primarily known for enhancing chemical stability under acidic conditions and improving permeability across cellular membranes. Carbamates are carbamic acid esters used in the prodrug approach to achieve first-pass and systemic hydrolytic stability. They are generally more enzymatically stable than the esters [22]. As a result, several natural phenolic compounds such as resveratrol, quercetin, and pterostilbene, have been developed using the carbamate prodrug approach. [25–29]. In designing a carbamate prodrug, a phenolic-OH group of a parent compound is linked to suitable non-toxic natural promoieties such as amino acids (Leu, Ile, Phe, Thr), polyols (glycerol), sugars (galactose) and polymers (polyethylene glycol) via an *N*-monosubstituted carbamate ester (-OC(O)NHR) linkage [25–29]. For promoieties without amino-functional groups such as glycerol, galactose, and polyethylene glycol, the amino group must be introduced by 3–4 more steps in the carbamate synthesis route. The primary amine was activated by reacting with bis(4-nitrophenyl) carbonate and then transesterification with a phenolic-OH group of a parent compound to give a carbamate prodrug. Amino acids are the most commonly used promoieties in carbamate prodrugs because they contain an amino-functional group readily for conjugation. However, it provided unsatisfactory absorption of the prodrug due to the high hydrophilicity from ionizable carboxylate groups, resulting in a negligible amount of carrier-mediated uptake [28]. Prodrugs containing amino acids with hydrophobic side chains were developed and demonstrated better permeability and absorption than the parent compound after oral administration to rats [25].

Gamma-aminobutyric acid (GABA) is a natural amino acid derivative found in microorganisms, plants, vertebrates and mammalians [30]. GABA can be actively transported to the brain by GABA transport (GAT)/betaine-GABA transporter [31]. In contrast, the basolateral GABA transporter and the proton-coupled amino acid transporter (hPAT1) are involved in GABA absorption in the intestine [32]. The neuronal GABA physiological functions include synaptic transmission modification, promoting neuronal growth and relaxation, and preventing insomnia and depression [33, 34]. Furthermore, anti-oxidant, anti-inflammation, anti-microbial, anti-hypertension, hepatoprotection, and intestinal protection were reported as GABA pharmacological properties on non-neuronal peripheral tissues and organs [34]. The FDA has approved GABA as a food ingredient [35]. As a result, the use of GABA as a promoiety could be safe.

In the present study, we designed and synthesized a novel GABA ethyl ester (GE) prodrug of curcumin, curcumin diethyl γ-aminobutyrate (CUR-2GE, Fig 1) via a carbamate linkage to improve prodrug stability under an acidic environment and lipophilicity. The physicochemical properties including solubility, partition coefficient, kinetic studies of hydrolysis and bioconversion in human plasma were investigated. In addition, the anti-neuroinflammatory effects of CUR-2GE were evaluated on LPS-stimulated BV-2 microglial cells and compared to curcumin.

## Materials

Curcumin was purchased from Shaanxi Kanglai Ecology Agriculture Co., Ltd. (Xi’an, China). GABA-ethyl ester and bis(4-nitrophenyl) carbonate were obtained from Tokyo Chemical Industry (TCI, Tokyo, Japan). The chemicals, solvents and reagents were ethyl acetate dichloromethane (DCM), hexanes (RCI, Labscan Bangkok, Thailand), 4-dimethylaminopyridine (DMAP) (Sigma-Aldrich, St. Louis, MO, USA), sodium sulfate (Merck, Darmstadt, Germany), acetonitrile (Burdick and Jackson, Ulsan, Korea). The chemicals and instruments used in the physicochemical properties, stability and anti-neuroinflammatory effect, including lipopolysaccharide (LPS) and 3-(4,5-dimethylthiazol-2-yl)-2,5-diphenyltetrazolium bromide (MTT), dimethyl sulfoxide (DMSO), Dulbecco’s Modified Eagle Medium (DMEM) without phenol red were purchased from Sigma-Aldrich (St. Louis, MO, USA). Potassium chloride, glacial acetic acid, monobasic potassium phosphate and sodium acetate anhydrous are supplied by Scharlau (Sentmenat, Spain). Acetonitrile was from Fisher Scientific (Seoul, Korea). An Elga Maxima 21F water purification system (Veolia Water Technologies, Wycombe, UK) was used to generate ultrapure water. Ethanol, sodium hydroxide and sodium lauryl sulfate (SLS) were purchased from Carlo Erba (Barcelona, Spain). Hydrochloric acid and n-octanol were purchased from QRëc (Auckland, New Zealand) and Panreac Quimica (Barcelona, Spain), respectively. Human plasma was provided from Nation Blood Center, Thai Red Cross Society (Bangkok, Thailand). BV-2 murine microglia were supplied by AcceGen Biotechnology (New Jersey, USA). DMEM with phenol red were obtained from Gibco, Thermo Fisher Scientific (Waltham, MA, USA). Fetal bovine serum was supplied by Merck Millipore (Burlington, MA, USA). L-Glutamine, penicillin and streptomycin were purchased from Caisson Labs (Smithfield, Utah, United States).

## Methods

### Synthesis and structural elucidation

The carbamate ester prodrug, CUR-2GE, was prepared in two steps: firstly, the primary amine of GABA ethyl ester was activated by reacting with bis(4-nitrophenyl) carbonate to produce the intermediate compound, ethyl 4-(((4-nitrophenoxy)carbonyl)amino)butanoate (GE-1). After that, the curcumin was conjugated with GE-1 by esterification reaction [28]. The chemical structures of CUR-2GE and GE1 were elucidated and confirmed by nuclear magnetic resonance spectroscopy (NMR) and high-resolution mass spectrometry (HRMS). ^1^H and ^13^C-NMR were performed on a Bruker Fourier 400 MHz (Bruker, Zuerich/Faellanden​, Switzerland) using CDCl_3_ as a solvent. The chemical shifts and coupling constants were measured in parts per million (ppm) and hertz (Hz), respectively. HRMS were operated on a MicrOTOF-QII Bruker time-of-flight high-resolution mass spectrometer coupled with an electrospray ion source (Bruker, Bremen, Germany).

#### Ethyl 4-(((4-nitrophenoxy)carbonyl)amino)butanoate (GE-1)

A solution of GABA-ethyl ester (0.685 g, 4.1 mmol) and DMAP (1.0 g, 8.2 mmol) in acetonitrile (20 mL) was added dropwise to a solution of bis(4-nitrophenyl) carbonate (1.37 g, 4.5 mmol) in acetonitrile (10 mL) and the resulting solution was stirred at 50°C for 3 h. The reaction mixture was then diluted in DCM (20 mL) and 0.5 N HCl (10 mL). The aqueous layer was extracted with DCM (3 × 10 mL) and all the organic fractions were collected, dried over sodium sulfate, filtered and evaporated under reduced pressure (Büchi Rotavapor R-200, Flawii, ST. Gallen, Germany). The obtained residue was purified by column chromatography on silica gel (Merck, Darmstadt, Germany) (hexane:EtOAc = 7:3) to obtain a colorless oil (920.87 mg, 75.68%). Thin-layer chromatography on a silica gel 60 G254 (Merck, Darmstadt, Germany) was used to monitor each eluent fraction. Rf; GE-1 = 0.22 and bis(4-nitrophenyl) carbonate = 0.58 (hexane:EtOAc = 7:3). ^1^H NMR (300 MHz, CDCl_3_) δ 8.27 (d, *J* = 9.0 Hz, 2H, H10), 7.34 (d, *J* = 9.1 Hz, 2H, H9), 5.46 (s, NH), 4.18 (q, *J* = 7.0 Hz, 2H, H2), 3.38 (q, *J* = 6.9 Hz, 2H, H6), 2.45 (t, *J* = 7.0 Hz, 2H, H4), 1.96 (p, *J* = 7.0 Hz, 2H, H5), 1.60 (s, OH), 0.90 (t, *J* = 6.8 Hz 3H, H1) (S1 Fig); HRMS (ESI) m/z calculated for (C_13_H_16_N_2_O_6_Na) 319.0901, found 319.0898 [M+Na^+^] (PPM = 0.94) (S2 Fig).

#### Curcumin monoethyl γ-aminobutyrate (CUR-1GE)

GE-1 (60 mg, 0.2 mmol) in 5 mL of DCM was added dropwise to a mixture of curcumin (184 mg, 0.5 mmol) and DMAP (48 mg, 0.4 mmol) in 10 mL of DCM. The reaction was stirred at 50°C for 24 h and 10 mL of 0.5 N HCl was added. The collected aqueous layer was extracted with DCM (3 × 10 mL). The combined organic solution was dried over sodium sulfate, concentrated under reduced pressure. The obtained residue was purified by column chromatography on silica gel (hexane:EtOAc = 6:4) to yield curcumin monoethyl γ-aminobutyrate (CUR-1GE, Fig 1) as an orange powder (76.60 mg, 72.88%). Rf; CUR-1GE = 0.28, curcumin = 0.55 and GE-1 = 0.7 (hexane:EtOAc = 6:4). ^1^H NMR (300 MHz, CDCl_3_) δ 7.62 (d, *J* = 15.8 Hz, 2H), 7.16 (dd, *J* = 9.9, 7.2 Hz, 2H, H4, 4׳), 7.16 (s, 1H, H6), 7.14 (d, *J* = 2.8 Hz, 1H, H9, 10, 10׳), 7.08 (s, 1H, H6׳) 6.95 (d, *J* = 8.2 Hz, 1H, H9׳), 6.54 (t, *J* = 15.3 Hz, 2H, H3, 3׳), 5.85 (s, 1H, H1), 5.30 (t, *J* = 6.0 Hz, NH), 4.18 (q, *J* = 7.1 Hz, 2H, H6׳׳), 3.97 (s, 3H, OCH_3_CHC), 3.91 (s, 3H, OCH_3_), 3.36 (q, *J* = 6.9 Hz, 2H, H2׳׳), 2.45 (t, *J* = 7.0 Hz, 2H, H4׳׳), 1.94 (p, *J* = 6.9 Hz, 2H, H3״), 1.61 (s, OH), 1.15–0.95 (t, *J* = 6.5 Hz, 3H, H7׳׳); ^13^C NMR (75 MHz, CDCl3) δ 184.38, 181.98, 173.25, 154.10, 151.91, 148.01, 146.85, 141.48, 141.07, 139.57, 133.60, 127.57, 124.05, 123.61, 123.03, 121.78, 121.00, 114.88, 111.43, 109.68, 101.51, 60.60, 55.97, 40.73, 31.48, 25.00, 14.24 (S3 and S4 Figs); HRMS (ESI) m/z calculated for (C_28_H_31_NO_9_Na) 548.1891, found 548.1864 [M+Na^+^] (PPM = 4.85) (S5 Fig). Chromatographic purity was 99.16% determined by ultra performance liquid chromatography (UPLC) (S6 Fig).

#### Curcumin diethyl γ-aminobutyrate (CUR-2GE)

Curcumin (62 mg, 0.169 mmol) and DMAP (81 mg, 0.676 mmol) were dissolved in 10 mL of DCM and added dropwise to GE-1 (226 mg, 0.761 mmol) in 5 mL of DCM. The reaction was stirred at 50°C overnight. The reaction mixture was added with 10 mL of 0.5 N HCl. The aqueous layer was extracted with DCM (3 × 10 mL). The combined organic solution was dried over sodium sulfate, concentrated under reduced pressure and purified by column chromatography on silica gel (hexane:EtOAc = 6:4) to yield CUR-2GE as yellow solid (54 mg, 47.41%). Rf; CUR-2GE = 0.11, CUR-1GE = 0.28, curcumin = 0.55 and GE-1 = 0.7 (hexane:EtOAc = 6:4). ^1^H NMR (300 MHz, CDCl_3_) δ 7.64 (d, *J* = 15.7 Hz, 2H, H4, 4׳), 7.14 (d, *J* = 6.7 Hz, 6H, H6, 9, 10, 6׳, 9׳, 10׳), 6.57 (d, *J* = 15.7 Hz, 2H, H3, 3׳), 5.88 (s, 1H, H1), 5.31 (t, *J* = 5.6 Hz, NH), 4.18 (q, *J* = 7.1 Hz, 4H, H6״), 3.94 (s, 6H, OCH_3_), 3.35 (q, *J* = 6.9 Hz, 4H, H2״), 2.44 (t, *J* = 7.0 Hz, 4H, H4״), 1.90 (p, *J* = 6.9 Hz, 4H, H3״), 1.61 (s, OH), 0.91 (t, *J* = 6.9 Hz, 6H, H7״); ^13^C NMR (75 MHz, CDCl_3_) δ 183.13, 173.25, 154.08, 151.93, 141.59, 140.07, 133.49, 124.07, 123.63, 121.11, 111.47, 101.75, 60.60, 55.97, 40.73, 31.48, 25.00, 14.24 (S7 and S8 Figs); HRMS (ESI) m/z calculated for (C_35_H_42_N_2_O_12_Na) 705.2635, found 705.2629 [M+Na^+^] (PPM = 0.85) (S9 Fig). Chromatographic purity was 96.18% determined by UPLC (S6 Fig).

### UPLC analysis

UPLC condition was modified from a previous report on an analysis of CDD [36]. The quantification of CUR-2GE, CUR-1GE and curcumin was performed on the Waters Acquity UPLC^TM^ H-Class system (Waters Corporation, MA, USA). The samples were separated on Acquity UPLC^TM^ BEH C18 1.7 μm, 2.1 x 50 mm column (Waters Chromatography Ireland Limited, Dublin, Ireland) at 33°C. The mobile phase consisted of 2%v/v acetic acid in water (A) and acetonitrile (B). The gradient program was used with the following profiles: initial A-B of 55:45 at 0 min; linear-gradient A-B of 20:80 from 0–2.7 min; isocratic A-B of 20:80 from 2.7–4.5 min; linear-gradient A-B of 55:45 from 4.5–5.0 min; isocratic A-B of 55:45 from 5.0–7.0 min. The flow rate was 0.3 mL/min, and the injection volume was 2 μL. The photodiode array detector was set at 400 nm (λ_max, CUR-2GE_ = 400.5, λ_max, CUR-1GE_ = 415.0, λ_max, Curcumin_ = 428.2). The Waters Empower^TM^ 3 software was used for system control and data processing. The retention times of curcumin, CUR-1GE and CUR-2GE were 1.6, 2.3 and 2.8 min, respectively (S4 Fig). The UPLC condition was applied for determination of the chromatographic purity and physicochemical properties of the synthesized compounds.

### Determination of physicochemical properties

#### Powder X-ray diffraction

The crystalline characteristics of CUR-2GE were determined using a powder x-ray diffractometer (PXRD) (Bruker, WI, USA) with Cu Kα radiation (λ = 1.5418 Å for combined K_α1_ and K_α2_) [18]. A 1 g of CUR-2GE was spread on a glass plate. The scanned angle range of XRD patterns was 2.0–50.0°. The scan rate, the voltage and the current of the X-ray generator were set at 2.4°/min, 40 kV and 15 mA, respectively.

#### Solubility

An excess amount of CUR-2GE (2 mg) was added to 2 mL of three solvents, water, phosphate buffer pH 4.5 and ethanol. The samples were sonicated at 25°C for 1 h and then placed on an orbital shaker at 100 rpm at 25°C for 1 h. The obtained mixture was then centrifuged at 25°C at 14,000 rpm for 10 min [37, 38]. The supernatant was analyzed using UPLC and the solubility of CUR-2GE in each medium was determined. Experiments were performed in triplicate. The solubility category was classified according to USP [39]. The percentage of CUR-2GE in the undissociated form at various pH values was calculated based on the Henderson-Hasselbalch equation [40] (see S1 Appendix).

For solubility mimicking gastrointestinal pH, an excess amount of CUR-2GE (1 mg) was added to a vial containing 2 mL of buffer pH 1.2, 4.5 or 6.8 with and without a surfactant (0.5% SLS) [41]. The mixture was sonicated for 1 h before orbital shaking at 37°C at 100 rpm for 1 h. After that, the mixture was centrifuged at 25°C at 14,000 rpm for 10 min prior to UPLC analysis. Experiments were performed in triplicate. The dose number (D_0_) at different media was determined and the BSC solubility class was assigned. (see S1 Appendix).

#### Partition coefficient

The partition coefficient of CUR-2GE was quantified using the shake flask method according to the guideline of OECD 107 with some modifications [17, 42]. A saturated mixture of n-octanol/water was firstly prepared at 25°C by stirring an equal volume of n-octanol/water for 24 h and left standing until complete separation. A 1 mg of CUR-2GE was dissolved in saturated n-octanol and water at volume ratios of 1:1, 1:2 and 2:1 in screw cap tubes. Experiments were run in duplicate. The tube was shaken through 180° over the transverse axis approximately 100 times in 5 min at room temperature (25°C) to reach equilibrium and phase distribution. The phases were then separated by centrifugation at 25°C at 5,500 rpm for 10 min. The organic phase was collected followed by aqueous phase centrifugation at 25°C at 14,000 rpm for 10 min. A small aliquot of the organic phase was then diluted with acetonitrile. CUR-2GE concentrations in each phase were analyzed by UPLC, and the P_o/w_ value was determined. In addition to water used as an aqueous phase, the partition coefficient between n-octanol and buffer at pH 4.5 and the P_o/buffer pH 4.5_ value were determined (see S1 Appendix).

### Chemical stability

Chemical stability of CUR-2GE was performed at 37°C in aqueous buffer solutions at pH 1.2, 4.5, 6.8 and 7.4. CUR-2GE (1 mg) was dissolved in DMSO (1 mL) and adjusted with the diluent in a 5-mL volumetric flask to obtain a stock solution at 200 μg/mL. The stock solution was diluted with a pre-incubated buffer in a vial at 37°C at an initial concentration of 10 μg/mL [26]. Each sample was incubated at 37°C at appropriate intervals and analyzed using UPLC. The experiment was performed in triplicate. The hydrolytic products were also identified by comparing chromatographic retention times to curcumin and CUR-1GE. Kinetic profiles of CUR-2GE and its hydrolytic products, including curcumin and CUR-1GE, were determined by plotting percent peak area vs. incubation time. The linear slope of the natural logarithm of concentrations against time was used to calculate the degradation rate (k) and half-life (t_1/2_) using the linear pseudo-first-order model (see S1 Appendix).

### Release study

The amount of curcumin released from CUR-2GE in human plasma was determined at 37°C at various time points. A stock solution of CUR-2GE (100 μg/mL) in the diluent was prepared and diluted with pre-incubated human plasma at 37°C for 5 min to obtain the final concentration of 5 μg/mL. The mixture was incubated at 37°C for 5, 10, 20, 30 and 60 min and each aliquot (300 μL) was added with 300 μL of acetonitrile to stop the reaction and further centrifugated at 4°C at 14,000 rpm for 30 min. The concentration of CUR-2GE in the supernatant was analyzed using UPLC [19, 26]. Experiments were carried out in triplicates. The initial time point (0 min) was prepared by adding 300 μL acetonitrile to the pre-incubated human plasma. The percent peak area of CUR-2GE and its hydrolytic products was plotted against incubation time. The k and t_1/2_ values were determined using the linear pseudo-first-order model.

### Identification of incomplete hydrolytic products of CUR-2GE

The incomplete hydrolytic products of CUR-2GE were identified in buffers (pH 1.2, 4.5, 6.8 and 7.4) and human plasma. For hydrolysis in the buffer solutions, the stock solution of CUR-2GE at 200 μg/mL was diluted with a pre-incubated buffer in a vial at 37°C to obtain a final concentration at 10 μg/mL. Samples in buffers pH 1.2 and 4.5 were subsequently incubated at 37°C for 24 h while those in buffers pH 6.8 and 7.4 were incubated at 37°C for 7 min. For hydrolysis in human plasma, the stock solution of CUR-2GE at 100 μg/mL in the diluent was prepared and diluted with pre-incubated human plasma at 37°C for 5 min to obtain a final concentration of 5 μg/mL. The sample was incubated at 37°C for 1 h and an aliquot of 300 μL was added with 300 μL of acetonitrile to stop the reaction. The reaction mixture was then centrifuged at 4°C at 14,000 rpm for 30 min. The supernatant was analyzed using UPLC-MS/MS described below. CUR-2GE, CUR-1GE and curcumin standards were prepared at 10 μg/mL in diluent (2% acetic acid in water: acetonitrile (80: 20, v/v)) and were subjected to UPLC-MS/MS analysis. The retention times and MS/MS spectra of CUR-2GE and its hydrolytic products in buffers and human plasma were compared with those of the standards. The UPLC-MS/MS approach was modified from previous studies [43, 44]. The MRM mode was used to select targeted compounds then the product ion scan mode was used to identify their fragmentation pattern.

### UPLC-MS/MS analysis

The chromatography was performed on Waters Acquity UPLC^TM^ system equipped with Waters Acquity UPLC^TM^ I-Class Binary Solvent system pump (Waters Corporation, MA, USA), with modification from the UPLC analysis mentioned above. The Acquity UPLC^TM^ I-Class Binary Solvent system pump can be set to deliver the mobile phase at a flow rate range of 0.010–2.000 mL/min. The separation of analytes was achieved by using an Acquity UPLC^TM^ BEH C18 1.7 μm, 2.1 x 50 mm column (Waters Chromatography Ireland Limited, Dublin, Ireland) at 33°C. The mobile phase consisted of 2%v/v acetic acid in water (A) and acetonitrile (B), with gradient elution at a flow rate of 0.175 mL/min. The gradient elution program was optimized as follows: an initial A-B of 55:45 at 0 min; linear-gradient A-B of 20:80 from 0–2.7 min; isocratic A-B of 20:80 from 2.7–4.5 min; linear-gradient A-B of 55:45 from 4.5–5.0 min; isocratic A-B of 55:45 from 5.0–7.0 min. The injection volume was 2 μL.

Mass spectrometric analysis of CUR-2GE, CUR-1GE and curcumin was achieved with MS/MS detection in a positive ion mode using a Waters Xevo^TM^ TQ-S, triple-quadrupole tandem mass spectrometer (Waters Corporation, Milford, Manchester, UK). Detection of the ions was carried out in the multiple reaction monitoring (MRM) by monitoring the transition at m/z 683>177 for CUR-2GE, m/z 526>177 for CUR-1GE and m/z 369>177 for curcumin with 100 ms dwell time for all compounds. Product ion scan was triggered at a threshold of 50. The parameters used for the electrospray source were as follows: capillary voltage 3.0 kV, cone voltage 20, 25 and 65 V for CUR-2GE, CUR-1GE and curcumin, respectively, desolvation temperature 300°C, desolvation gas flow 800 L/h, cone gas flow 150.0 L/h and nebulizer flow 7.0 bar. The following conditions were set for the quadrupoles of the Xevo TQ-S spectrometer: LM1 resolution of 3.0, HM1 resolution of 15.0, ion energy 1 at 0.5, collision energy at 20 eV, LM2 resolution of 3.0, HM2 resolution of 15.0 and ion energy 2 at 0.5. System control, data acquisition, and data processing were performed using Waters MassLynx^TM^ software (Version 4.1 SCN950).

### *In vitro* cellular uptake

BV-2 microglial cells were plated in a 96-well plate (Costar, NY, USA) at a density of 10,000 cells/well for 24 h [13]. The cells were incubated in DMEM media without phenol red for 4 h in the presence of curcumin and CUR-2GE at concentrations of 20 μM and 100 μM. Then, the sample was washed with PBS and the fluorescence intensity was measured using a fluorescence microscope (Olympus IX51 inverted microscope, Tokyo, Japan).

### Anti-inflammatory effects and molecular mechanism

#### Cytotoxicity

BV-2 microglial cells were cultured in DMEM supplemented with 10% fetal bovine serum, 2 mM L-glutamine, 1% penicillin/streptomycin (100 units/mL penicillin and 100 μg/mL streptomycin) at 37°C in 5% CO_2_. BV-2 microglial cells were plated in a 24-well plate at a density of 2 × 10^5^ cells/well for 24 h [45]. Various concentrations at 0, 1.25, 2.5, 5, 10, or 20 μM of curcumin or CUR-2GE were determined for a non-toxic concentration. After 24 h, cell viability was assessed by an MTT assay. The media was removed and replaced with an MTT solution in PBS (0.5 mg/mL). After 3-h incubation, the MTT solution was discarded, and formazan crystals formed after the reaction was dissolved in DMSO. The absorbance was then read at 540 nm using a microplate reader (CLARIOstar^®^, BMG Labtech, Ortenberg, Germany). The percentage of cell viability was expressed relative to the control cells. In addition, the cell viability of BV-2 microglial cells treated with LPS and a combination of LPS and the test compounds was also determined.

#### Anti-inflammatory effects on LPS-stimulated BV-2 microglial cells

BV-2 microglial cells were plated in a 24-well plate at a density of 2 × 10^5^ per well for 24 h. The cells were pre-incubated for 12 h with curcumin (10 μM), CUR-2GE (10 μM), medium (DMEM), or a vehicle (0.5% DMSO). The cells were then induced with 1 μg/mL LPS for 24 h [45]. The culture medium samples were collected and analyzed for nitric oxide (NO) and cytokines (TNF- and IL-6) using NO and enzyme-linked immunoassay (ELISA) assays, respectively.

#### Nitric oxide assay

Nitric oxide (NO) production was determined by measuring nitrite, the NO metabolite, using Griess reagent [46]. Briefly, the culture medium (100 μL) was added with 1% sulfanilamide (50 μL) and incubated for 5 min. Then, a solution of 0.1% *N*-(1-naphthyl)ethylenediamine dihydrochloride (50 μL) was added and incubated for 5 min. The absorbance was measured at 520 nm using the microplate reader, and the nitrite concentration was calculated against a calibration curve using NaNO₂ as a standard.

#### Determination of TNF-α and IL-6

Quantification of TNF-α and IL-6 was performed using commercially available ELISA kits according to the manufacturer’s protocol (BioLegend, San Diego, CA, USA). The absorbance was measured at 450 nm using the microplate reader, and the amount of TNF-α and IL-6 was determined against their corresponding standard curves of TNF-α and IL-6.

#### Statistics

All experiments were performed in triplicate unless otherwise stated with means ± standard deviation values. The differences between groups were statistically analyzed using one-way ANOVA followed by the Bonferroni post hoc test. The *p*-value < 0.05 was considered to be statistically significant.

## Results and discussion

CUR-2GE was designed and synthesized to improve the physicochemical properties of curcumin and accordingly enhance anti-neuroinflammation activity. The carbamate linkage was selected to increase stability at acidic pH while GE served as the pro-moiety or carrier to improve lipophilicity. The synthesized CUR-2GE was characterized and determined for the physicochemical properties including solubility, partition coefficient, stability and bioconversion in human plasma in the simulated gastrointestinal fluids. *In vitro* cellular uptake and anti-inflammatory effects of CUR-2GE on LPS-stimulated BV-2 microglial cells were also investigated.

### Synthesis

The CUR-2GE prodrug was synthesized via two steps as shown in Fig 1. In the first step, the free amino group of GABA-ethyl ester reacted with bis(4-nitrophenyl) carbonate in the presence of 4-(dimethylamino)-pyridine (DMAP) as a catalyst to produce the activated 4-nitrophenyl carbamate, GE-1. In the last step, curcumin in excess was added to GE-1, and the designed CUR-2GE prodrug was formed in a medium yield of 47.41%. Alternatively, in the synthesis of CUR-1GE, GE-1 in excess was added to curcumin. The intermediate CUR-1GE was obtained in a high yield of 72.88%.

### Determination of physicochemical properties

#### Powder X-ray diffraction

PXRD was performed to evaluate the crystallinity of CUR-2GE. The PXRD spectrum of CUR-2GE exhibited several intense peaks (S10 Fig), indicating that the synthesized CUR-2GE powder was in a crystalline form.

#### Solubility

In this study, the solubility of CUR-2GE in common vehicles for formulation including water and ethanol was determined. Since CUR-2GE is a prodrug that can be hydrolyzed in an aqueous solution, the solubility in buffer pH 4.5 was also conducted to avoid significant hydrolysis as CUR-2GE was relatively more stable under acidic pH (see Stability section below). In addition, the short saturation time in the solubility test, 1-h sonication followed by 1-h shaking, was employed to avoid hydrolysis of CUR-2GE. The degradation of CUR-2GE in all media except for the buffer at pH 1.2 with SLS is less than 10%, which falls within the ICH limit for degradation of the tested substance at 10% [47], indicating the validity of the solubility results.

In water and buffer pH 4.5, CUR-2GE solubility was less than 0.05 μg/mL, while in ethanol, the solubility increased to 17.85 μg/mL (Table 1). According to USP, the solubility classification of CUR-2GE in water, buffer pH 4.5 and ethanol is designated as practically insoluble (<0.1 mg/mL), similar to that of curcumin [17, 48, 49]. The results suggest that ethanol would be an alternative solvent or co-solvent for the formulation of CUR-2GE.

**Table 1:** Solubility of CUR-2GE, BCS solubility classification and partition coefficients (Log P).

Condition	Solubility (μg/mL)					Log P	
	Water	Ethanol	Buffers			Water	Buffer pH 4.5
			pH 1.2	pH 4.5	pH 6.8		
25°C	<LOQ	17.85 ± 1.76	ND	<LOQ	ND	3.57 ± 0.19	3.43 ± 0.14
37°C	ND	ND	<LOQ	0.64 ± 0.51	0.34 ± 0.50	ND	ND
37°C +0.5%SLS	ND	ND	NV	23.35 ± 0.18	148.12 ± 38.53	ND	ND
D_0_ x 10^3^	ND	ND	74.16	5.80	10.89	ND	ND

Solubility and partition coefficient presented as mean ± SD; <LOQ refers to less than 0.05 μg/mL; ND, not determined; NV, not valid because more than 10% of hydrolytic products of CUR-2GE was observed (S1 Table).

D_0_ calculated using M_0_: the highest dose strength of capsule (927 mg), C_s_: the saturation solubility of CUR-2GE at 37°C without 0.5%SLS (mg/mL) and V_0_: the initial gastric volume (250 mL).

In the biopharmaceutics classification system (BCS), the solubility of drug substances is conducted according to the international guidelines [47, 50] using a saturated solubility test at pH 1.2–6.8, 37°C to mimic pH in the gastrointestinal tract [51–53]. As shown in Table 1, CUR-2GE had low solubility (< 1 μg/mL) and high D_0_ values (> 1) in buffers at all tested pH. However, SLS significantly enhanced CUR-2GE solubility, particularly at pH 6.8, possibly due to the various effects of SLS in reducing surface tension, increasing wettability and forming micelles in the medium [54]. Physiologically, CUR-2GE solubility can be improved in the intestinal tract with endogenous surfactants such as bile salts and lipids. In buffer pH 1.2 with SLS, CUR-2GE was unstable with more than 10% degradation, leading to invalid solubility results (S11 Fig and S1 Table). SLS accelerated CUR-2GE degradation at buffer pH 1.2 is probably due to a process known as micellar catalysis [55–57].

#### Partition coefficient

The Log P_o/w_ and Log P_o/buffer pH 4.5_ values of CUR-2GE were found to be 3.57 and 3.43 as summarized in Table 1. It is of note that the amount of CUR-2GE in aqueous phases was not detectable, and therefore the LOQ of 0.05 μg/mL was used for Log P calculation. The Log P values in both conditions are more than 3, indicating that CUR-2GE is a lipophilic drug with good passive absorption [58]. The Log P_o/w_ of CUR-2GE is significantly greater than the previously reported values of curcumin ranging from 2.19 to 3.29 [18, 59–61]. The increased log P confirmed that the presence of the GE-promoities at both phenolic groups of curcumin contributed to the higher lipophilicity of CUR-2GE as designed.

### Stability and release study

**Fig 2:**
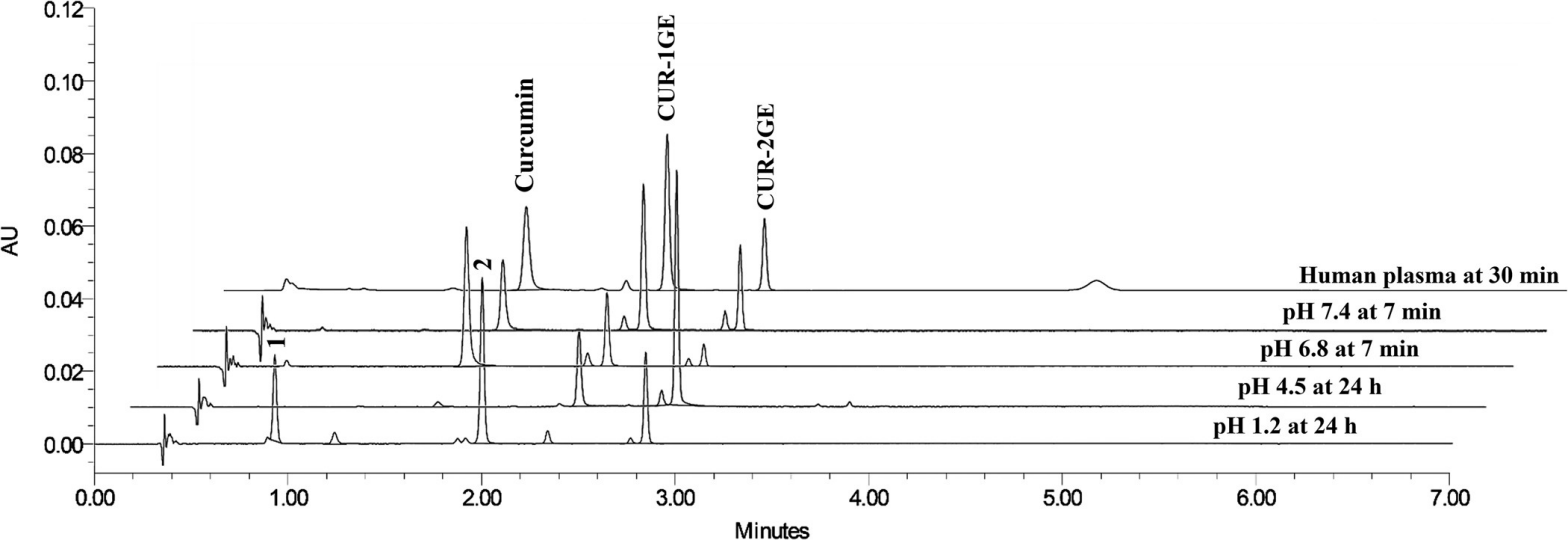
Representative chromatograms of CUR-2GE incubated at 37°C in buffers at pH 1.2, 4.5, pH 6.8, pH 7.4, and human plasma for 24 h, 24 h, 7 min, 7 min, and 30 min, respectively. The retention times of curcumin, CUR-1GE and CUR-2GE were 1.6, 2.3 and 2.8 min, respectively, and the retention times of major unknown compounds 1 and 2 were 0.9 and 2.0 min, respectively.

Hydrolysis of CUR-2GE was investigated in buffers (pH 1.2, 4.5, 6.8 and 7.4) and human plasma representing the pH of the gastrointestinal system and blood. Representative chromatograms of CUR-2GE at different pH are displayed in Fig 2. CUR-2GE was observed at a retention time of 2.8 min, two known products (curcumin and CUR-1GE at retention times of 1.6 and 2.3 min, respectively) and two major unknown compounds (compounds 1 and 2 at retention times of 0.9 and 2.0 min, respectively).

**Fig 3:**
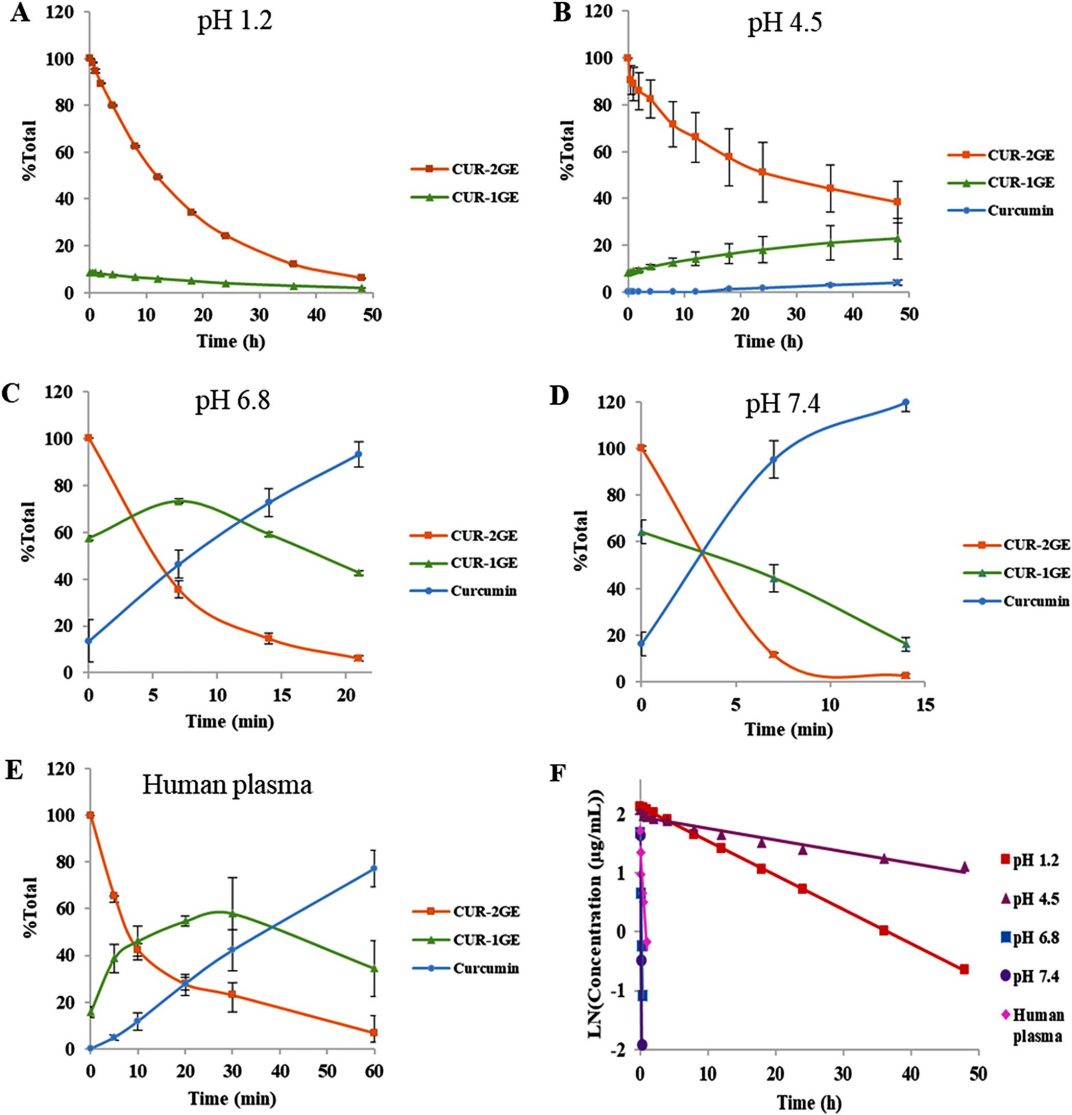
Chemical stability of CUR-2GE in various buffers and human plasma. Kinetic profiles of CUR-2GE and its hydrolytic products in buffers and human plasma: (A) pH 1.2, (B) 4.5, (C) 6.8, (D) 7.4 and (E) human plasma. Data of CUR-2GE and its hydrolytic products are expressed as the percentage of the initial peak area of CUR-2GE (%Total). (F) Pseudo-first-order kinetic plots of CUR-2GE hydrolysis in buffers and human plasma.

Kinetic profiles of CUR-2GE, the released CUR-1GE and curcumin in buffers at pH 1.2, 4.5, 6.8, 7.4 and human plasma are shown in Fig 3A–3E, respectively. The hydrolysis data of CUR-2GE in all tested media fitted well with the pseudo-first-order kinetic with r^2^ values close to 0.9 (Fig 3F and Table 2) and the kinetic parameters (k and t_1/2_) are summarized in Table 2. The k values of CUR-2GE were in the following order: pH 7.4 > 6.8 > 1.2 > 4.5 with the hydrolysis rate of CUR-2GE in buffer pH 7.4 higher than pH 6.8, 1.2 and 4.5 by 1.94, 262 and 778 folds, respectively. Consistency, the half-life value (t_1/2_) was in reverse to the k values as in the following order: pH 4.5 > 1.2 > 6.8 > 7.4.

**Table 2:** Pseudo-first-order kinetic parameters for hydrolysis of CUR-2GE at 37°C in buffers at pH 1.2, 4.5, 6.8 and 7.4 and human plasma.

pH	k (h^-1^)	t_1/2_ (h)	r^2^
1.2	0.0583 ± 0.0007	11.88 ± 0.14	0.9998 ± 0.0001
4.5	0.020 ± 0.004	36.41 ± 8.53	0.9503 ± 0.0328
6.8	7.89 ± 0.54	0.09 ± 0.01	0.9977 ± 0.0012
7.4	15.31 ± 0.70	0.045 ± 0.002	0.9868 ± 0.0018
Human plasma	1.76 ± 0.30	0.40 ± 0.08	0.8937 ± 0.0946

The chemical stability of CUR-2GE in human plasma was determined to confirm the bioconversion of CUR-2GE to curcumin. CUR-2GE completely released curcumin in human plasma via CUR-1GE at the first hour with the t_1/2_ value of 0.40 h (Fig 3E), consistent with previous reports on the half-life of carbamate prodrugs in the blood ranging from 0.17 to 1 h [28, 29]. CUR-2GE was converted to curcumin with the rate in human plasma approximately 8.7-fold lower than that in buffer pH 7.4, suggesting that plasma protein may stabilize CUR-2GE [62]. However, comparing stability between CUR-2GE and curcumin in human plasma, the t_1/2_ value of CUR-2GE in human plasma was 0.40 h which was significantly less than the half-life of curcumin in human plasma previously reported at 8 h [63]. These results infer that the selected carbamate bond connecting the parent curcumin to the promoiety was rapidly hydrolyzed to yield the intermediate CUR-1GE and curcumin. Thus, the CUR-2GE carbamate prodrug may prolong the plasma exposure of curcumin, resulting in a higher amount of curcumin for exerting its biological effects.

**Fig 4:**
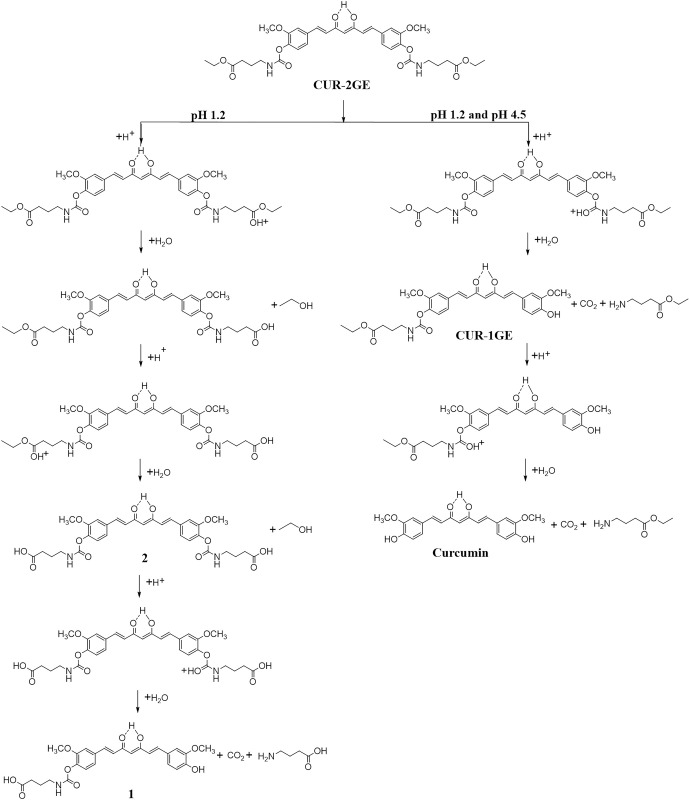
Proposed acid-catalyzed hydrolysis mechanisms of CUR-2GE in buffers at pH 1.2 and 4.5.

At acidic pH 1.2, two peaks of the unknown hydrolytic products (1 and 2) were observed, possibly due to acid-hydrolysis of the ester group of GABA with the plausible mechanism shown in Fig 4 [22, 64]. However, these unknown compounds are absent at pH 4.5. The higher stability of CUR-2GE in buffer pH 4.5 possibly due to the 2 H-bond stabilization, resulting from dimer formation between the syn carbamate groups of CUR-2GE and an acetate ion in the buffer [22, 65–67].

**Fig 5:**
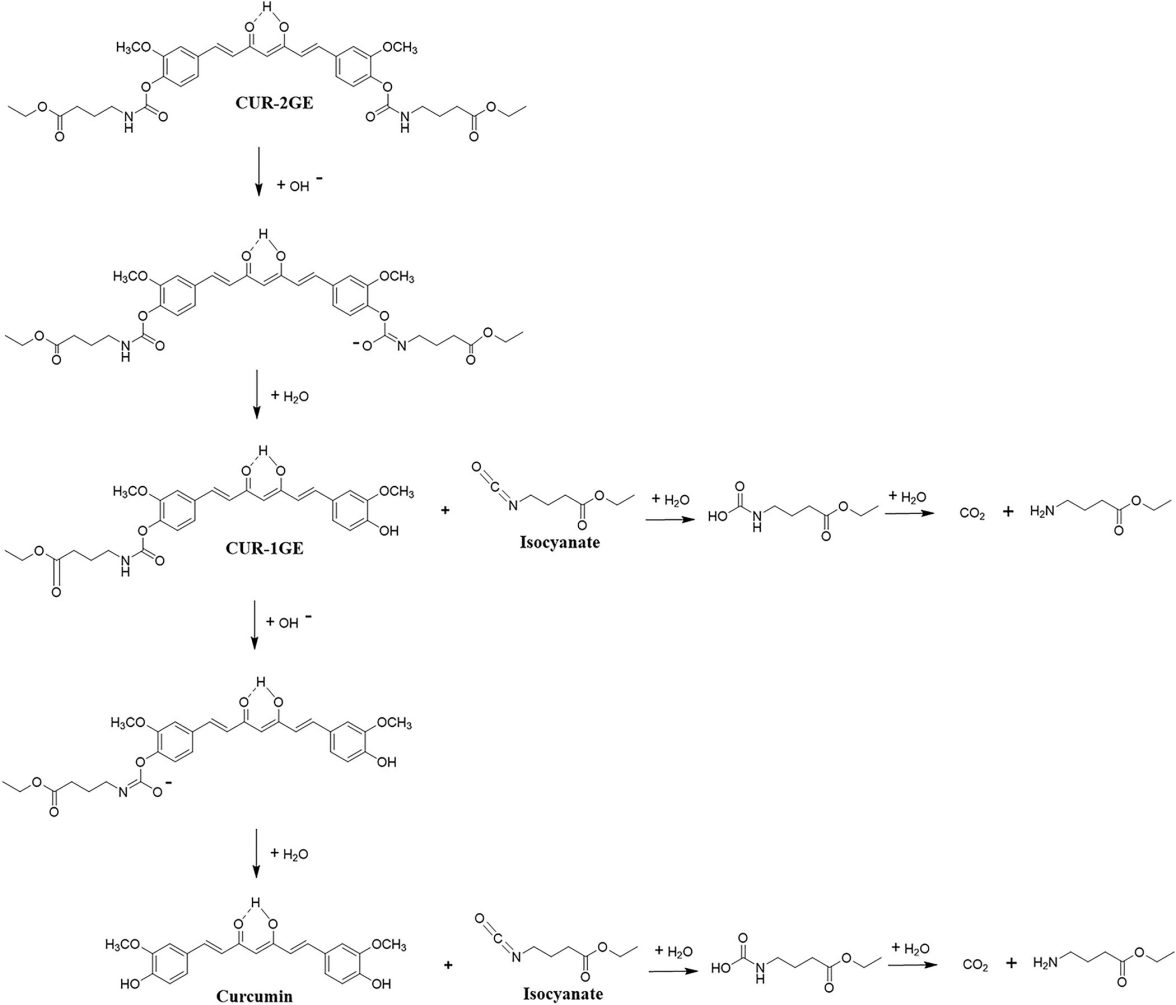
Proposed hydrolysis mechanisms of CUR-2GE in buffers at pH 6.8 and 7.4.

At pH 6.8 and 7.4 close to the neutral pH, CUR-2GE was hydrolyzed to the parent curcumin via the CUR-1GE intermediate (Fig 5). The degradation mechanism may be due to the hydrolysis of carbamates through deprotonation and elimination process giving two intermediates, CUR-1GE and isocyanate. The CUR-1GE was further hydrolyzed via the same mechanism. The isocyanate intermediate rapidly reacted with water and further decomposed generating carbon dioxide. The isocyanate formation may account for the significant increase in hydrolytic rate previously observed with aromatic N-monosubstituted carbamate esters [68].

### Mass fragmentation of CUR-2GE, CUR-1GE and curcumin

The MS parameters were tuned in a positive electrospray ionization mode. The mass fragmentation patterns of CUR-2GE, CUR-1GE and curcumin standards were shown in S12 Fig (see Supplementary section). The precursor ions of CUR-2GE, CUR-1GE and curcumin at m/z 683, 526 and 369, respectively, were fragmented in the collision cell to the same predominant product ion at m/z 177. Hence, the multiple reaction monitoring (MRM) mode was adopted for analysis with the transitions of m/z 683>177 for CUR-2GE, m/z 526>177 for CUR-1GE and m/z 369>177 for curcumin.

### Identification of incomplete hydrolytic products of CUR-2GE

**Fig 6:**
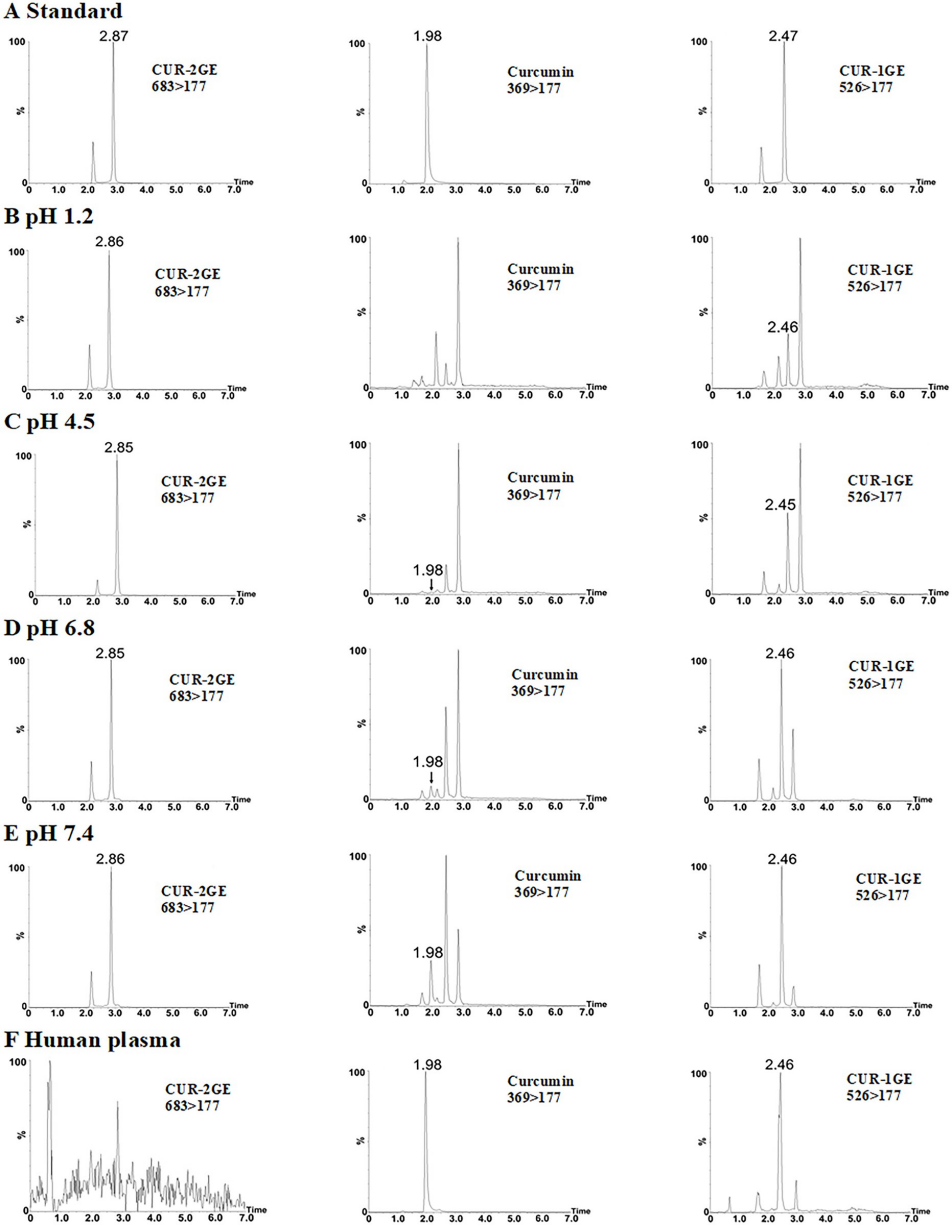
UPLC-MS/MS chromatograms of CUR-2GE, curcumin, CUR-1GE. (A) CUR-2GE, curcumin and CUR-1GE standards at 10 μg/mL, CUR-2GE incubated at 37°C in buffers at (B) pH 1.2 for 24 h, (C) pH 4.5 for 24 h, (D) pH 6.8 for 7 min, (E) pH 7.4 for 7 min and (F) human plasma for 1 h. The peak of CUR-2GE, curcumin and CUR-1GE was shown at the retention times about 2.87, 1.98 and 2.47, respectively. The peak with retention times of 2.18 and 1.69 min represented tautomers of CUR-2GE and CUR-1GE, respectively, as they exhibited the same transition ions. Other peaks were generated from an insource fragmentation.

The retention times and product ions of CUR-2GE, CUR-1GE and curcumin are summarized in Table 3. The UPLC-MS/MS chromatogram showed the retention times in the order of CUR-2GE, curcumin and CUR-1GE about 2.87, 1.98 and 2.47 min, respectively (Fig 6). The peak with a retention time of 2.18 min and 1.69 min represented a tautomer of CUR-2GE and CUR-1GE, respectively, as it exhibited the same transition ion, which was similar to curcumin and other curcumin prodrugs [69–72].

**Table 3:** UPLC-MS/MS results of CUR-2GE and its hydrolytic products after incubation.

Analyte	Source	Retention time (min)	Formula	MRM transition (m/z)	Cone voltage (V)	Collision energy (eV)	Product ions (m/z)
CUR-2GE Curcumin CUR-1GE	Standard	2.87	C_35_H_42_N_2_O_12_	683.25>177.10	20	20	177.24, 245.08, 285.05, 369.15, 526.04
	Standard	1.98	C_21_H_20_O_6_	369.15>177.10	65	20	177.09,245.08, 285.01
	Standard	2.47	C_28_H_31_NO_9_	526.15>177.10	25	20	177.12, 244.90, 285.16, 369.28
CUR-2GE	pH 1.2	2.86	C_35_H_42_N_2_O_12_	683.25>177.10	20	20	176.53, 369.41, 526.62
	pH 4.5	2.85	C_35_H_42_N_2_O_12_	683.25>177.10	20	20	177.43, 245.45, 285.02, 369.26, 526.46
	pH 6.8	2.85	C_35_H_42_N_2_O_12_	683.25>177.10	20	20	177.42, 244.77, 368.91, 526.73
	pH 7.4	2.86	C_35_H_42_N_2_O_12_	683.25>177.10	20	20	177.06, 285.07, 368.95, 526.45
	Human plasma	ND	C_35_H_42_N_2_O_12_	683.25>177.10	20	20	ND
Curcumin	pH 1.2	ND	C_21_H_20_O_6_	369.15>177.10	65	20	ND
	pH 4.5	1.98	C_21_H_20_O_6_	369.15>177.10	65	20	177.04, 244.80, 285.48
	pH 6.8	1.98	C_21_H_20_O_6_	369.15>177.10	65	20	177.12, 245.54, 285.18
	pH 7.4	1.98	C_21_H_20_O_6_	369.15>177.10	65	20	177.40, 245.01, 284.94
	Human plasma	1.98	C_21_H_20_O_6_	369.15>177.10	65	20	177.12, 245.23, 285.18
CUR-1GE	pH 1.2	2.46	C_28_H_31_NO_9_	526.15>177.10	25	20	176.98, 246.19, 285.48, 368.57
	pH 4.5	2.45	C_28_H_31_NO_9_	526.15>177.10	25	20	177.29, 245.02,288.07, 367.81
	pH 6.8	2.46	C_28_H_31_NO_9_	526.15>177.10	25	20	177.27, 249.93, 285.25, 371.52
	pH 7.4	2.46	C_28_H_31_NO_9_	526.15>177.10	25	20	177.15, 244.79, 284.26, 368.80
	Human plasma	2.46	C_28_H_31_NO_9_	526.15>177.10	25	20	178.88, 368.87

ND = not detected.

**Fig 7:**
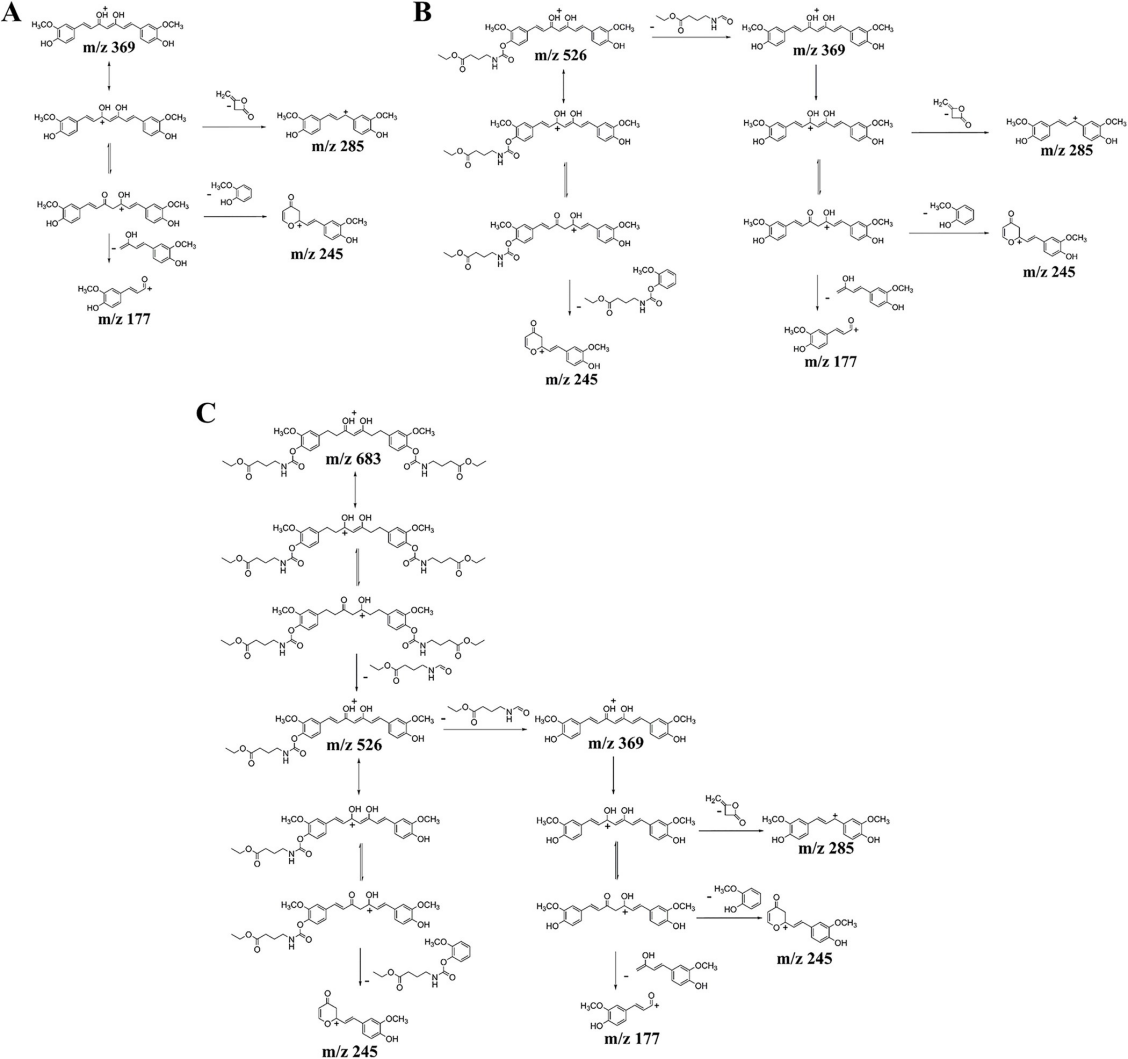
Proposed mass fragmentations of (A) curcumin, (B) CUR-1GE and (C) CUR-2GE.

The MS/MS spectra of curcumin showed several product ions at m/z 177, 245 and 285 (Table 3). CUR-1GE lost a mono-ethyl γ-aminobutyrate moiety leading to the formation of curcumin ion at m/z 369 and several product ions at m/z 177, 245 and 285 of curcumin. The MS/MS spectrum of CUR-2GE showed the loss of a mono-ethyl γ-aminobutyrate moiety to form a line peak at m/z 526, subsequently identified as CUR-1GE. In addition, CUR-2GE could lose two functional groups of ethyl γ-aminobutyrate resulting in the product ion with m/z 369, subsequently identified as curcumin. The fragmentations of curcumin, CUR-1GE and CUR-2GE are proposed in Fig 7.

The UPLC-MS/MS of curcumin in buffers at pH 4.5, 6.8, 7.4 and human plasma showed the retention time at 1.98 min consistent with the curcumin standard (Fig 6). The MS/MS spectra demonstrated the product ions at m/z 177, 245 and 285 (Table 3), confirming that the peak at 1.98 min was curcumin. In a similar analysis, the retention time about 2.46 min was consistent with the CUR-1GE standard (Fig 6). The product ions generated at m/z 177, 245, 285 and 369 in buffers and m/z 178 and 369 in human plasma confirmed the peak at 2.46 as CUR-1GE (Table 3).

Curcumin was not detected in acidic pH 1.2. CUR-1GE was found at all buffer pH levels and in human plasma, indicating that CUR-2GE was incompletely hydrolyzed and CUR-1GE, an intermediate of CUR-2GE, was formed before the parent curcumin.

### *In vitro* cell uptake in BV-2 microglial cells

**Fig 8:**
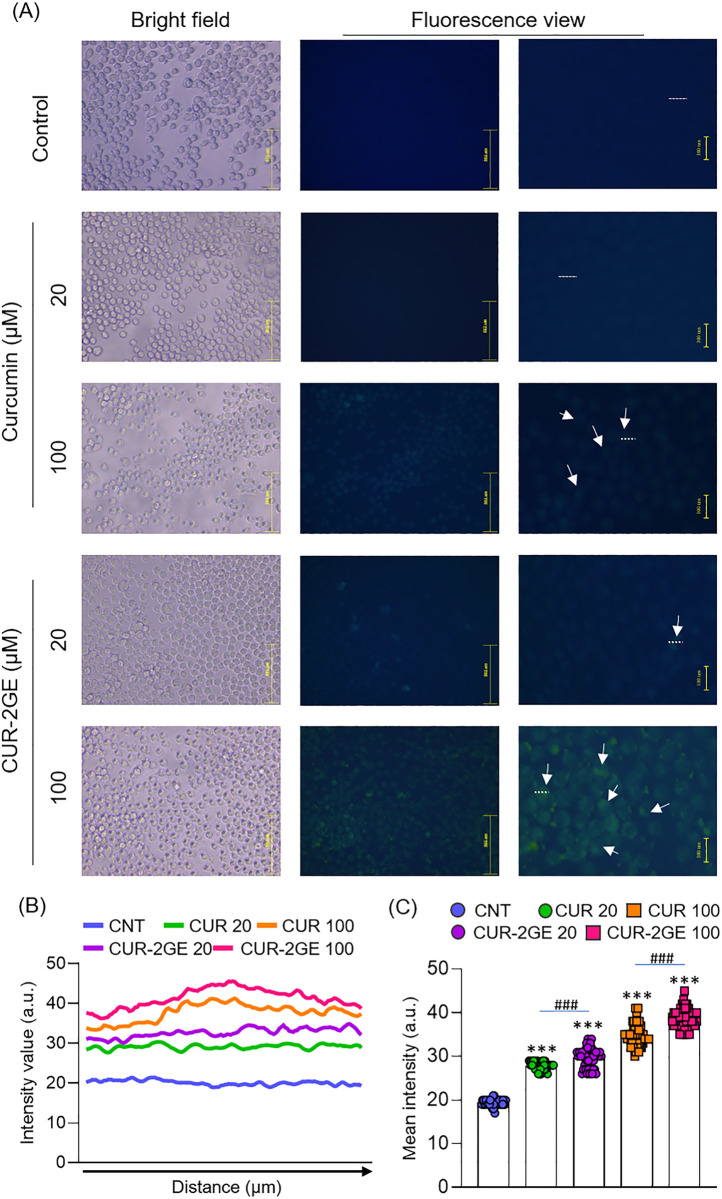
*In vitro* cellular uptake of curcumin and CUR-2GE. (A) Representative images of curcumin and CUR-2GE uptake in BV-2 microglial cells. The scale bars correspond to 502 μm (center) and 100 μm (right). The fluorescence intensity was further analyzed using ImageJ software. (B) Bar graph showing the fluorescent intensity of a single cell along the dotted line and (C) total average fluorescence intensity of 50 cells. The data in Fig 8C are expressed as mean ± SD (n = 50). ***p <0.001, control vs other treatments, ###p <0.001, Curcumin vs CUR-2GE.

To assess the uptake of CUR-2GE to microglial cells, the fluorescence intensity of curcumin or CUR-2GE in BV-2 microglial cells was monitored under a fluorescence microscope after 4-h incubation. As shown in Fig 8, both curcumin and CUR-2GE were evenly distributed in the cells (Fig 8A). The cells treated with CUR-2GE had higher fluorescence intensity than curcumin (Fig 8B and 8C), indicating the more cellular uptake of CUR-2GE. It was possibly due to its higher lipophilicity and consequently better cell penetration via passive transport. In addition, the presence of GABA-ethyl ester in the CUR-2GE may interact with GABA transporters commonly found in neuronal microglial cells, facilitating drug uptake into BV-2 microglial cells via active transport [73, 74].

### Anti-inflammatory effect on BV-2 microglial cells

#### Cytotoxicity

**Fig 9:**
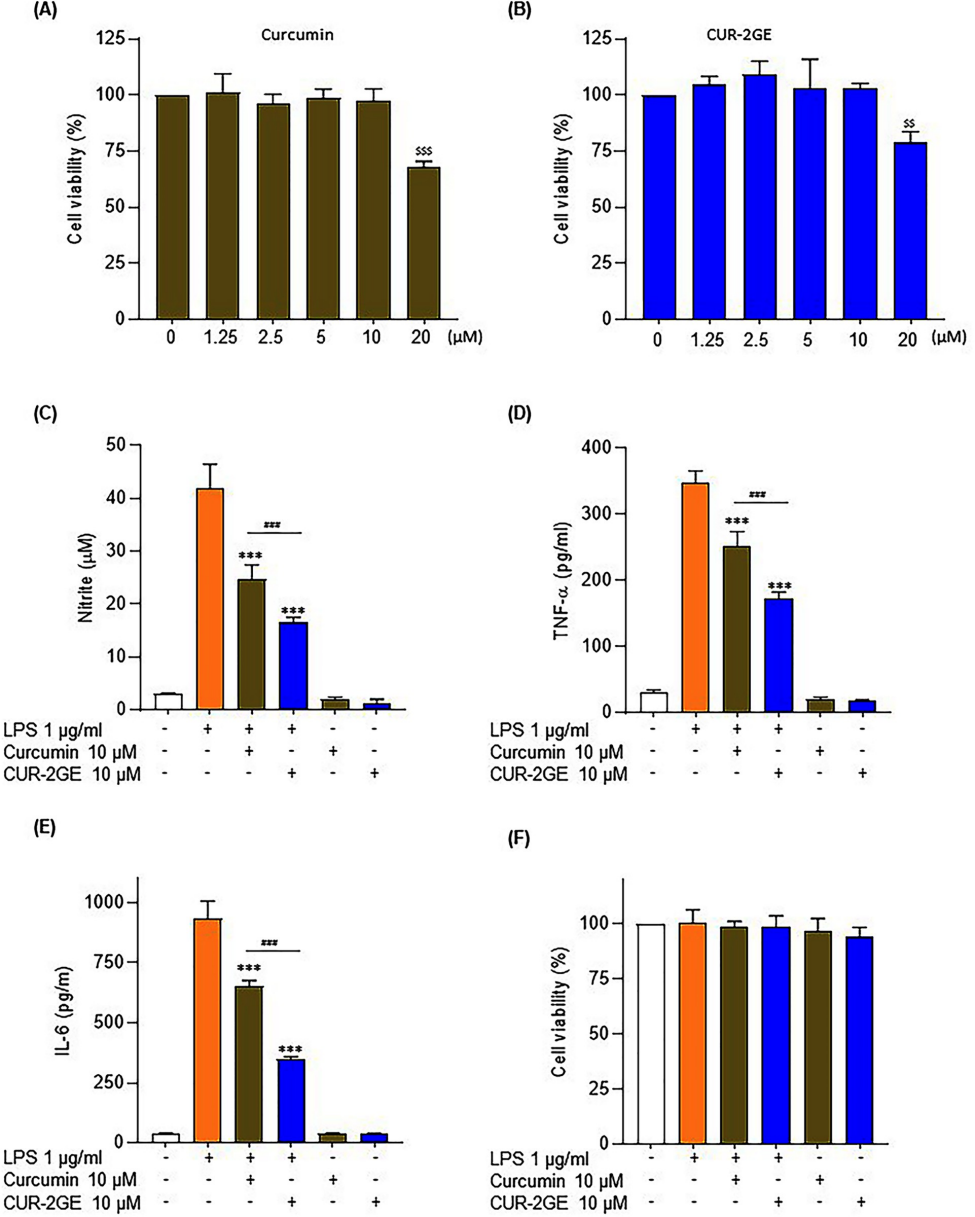
Effects of curcumin and Cur-2GE on cytotoxicity and secretion of pro-inflammatory mediators in LPS-stimulated BV-2 microglial cells. (A-B) Cytotoxicity, (C) NO, (D) TNF-α, (E) IL-6 and (F) Cell viability. Data are expressed as mean ± SD of three independent experiments. $ $p < 0.01, $ $ $p < 0.001 compared to the control group. ***p < 0.001 compared to the LPS group. ###p < 0.001 significant difference between curcumin and CUR-2GE groups. The differences were analyzed by one-way ANOVA followed by the Bonferroni post hoc test.

The cytotoxicity of curcumin and CUR-2GE was determined to identify their non-toxic concentrations. It was found that curcumin and CUR-2GE at concentrations ≤ 10 μM had no cytotoxicity and were considered as non-toxic concentrations (Fig 9A and 9B). Thus, the highest non-toxic concentration at 10 μM was used for subsequent cell-based assays.

#### Effects on NO, TNF-α and IL-6 levels

**Fig 10:**
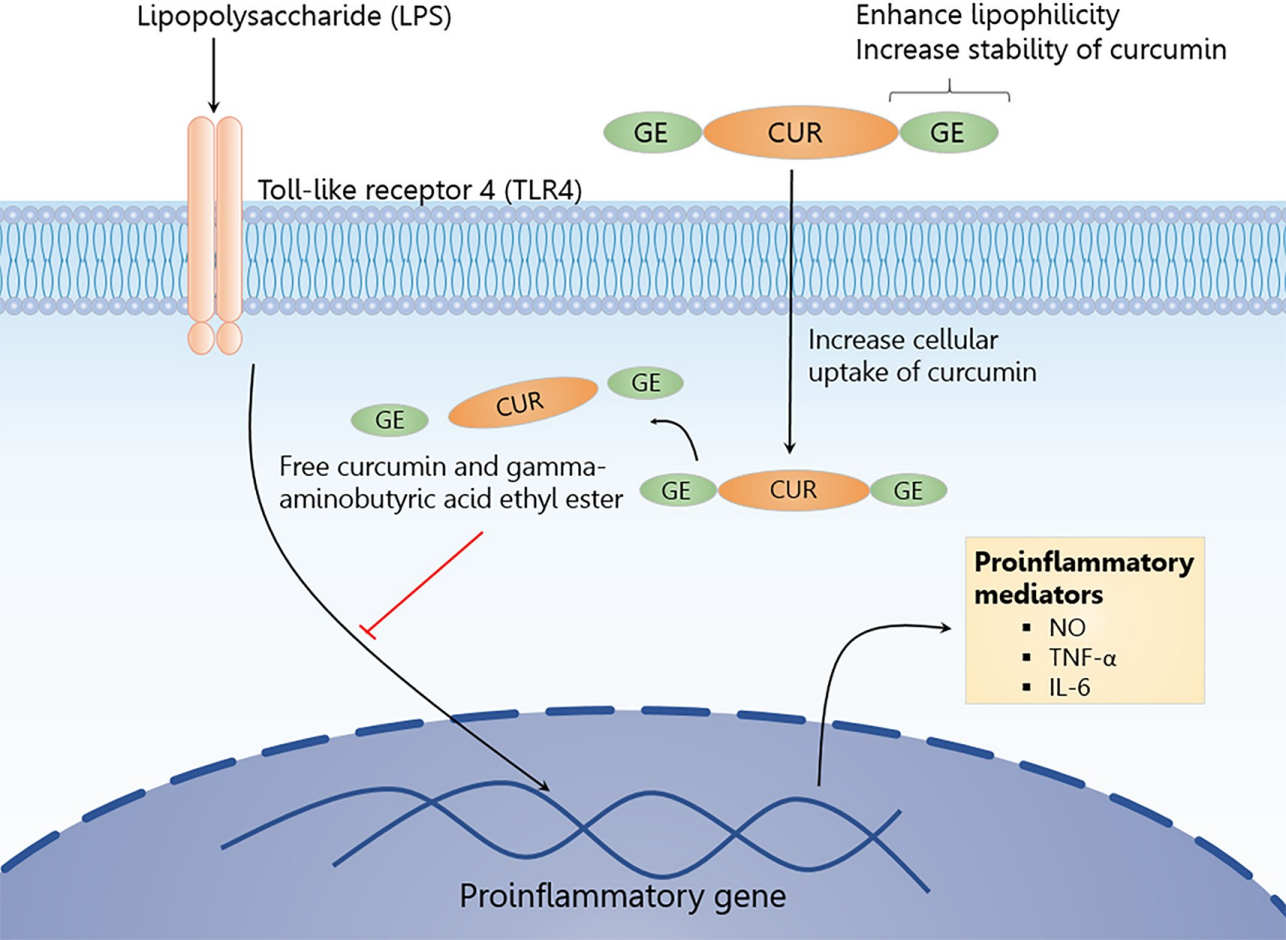
Proposed mechanism of CUR-2GE in LPS-stimulated BV-2 microglial cells. CUR-2GE with improved stability and lipophilicity profiles enhanced cellular uptake of curcumin, leading to greater anti-inflammatory effects in LPS-stimulated BV-2 cells. CUR, curcumin; GE, gamma-aminobutyric acid ethyl ester; IL-6, interleukin 6; LPS, lipopolysaccharide; NO, nitric oxide; TNF-α, tumor necrosis factor α.

The anti-neuroinflammatory effects of CUR-2GE on the secretion of pro-inflammatory mediators (NO, TNF-α and IL-6) were investigated in LPS-stimulated BV-2 microglial cells. LPS at 1 μg/mL was sufficient to robustly increase NO, TNF-α and IL-6 without affecting cell viability (Fig 9C–9F). CUR-2GE inhibited NO production and cytokine releases (TNF-α and IL-6) to a greater extent than curcumin in LPS-stimulated BV-2 microglial cells (Fig 9C–9E). Our results are consistent with the previous suggestion on the activity of curcumin against LPS-induced BV-2 microglial cells [10]. CUR-2GE had more significant anti-neuroinflammatory activity than curcumin, possibly due to the improved physicochemical properties via the prodrug approach. In summary, CUR-2GE significantly enhanced anti-neuroinflammatory activity and increased inhibition of pro-inflammatory mediators in the LPS-stimulated BV-2 microglial cell model (Fig 10).

## Conclusions

In this study, CUR-2GE, a new carbamate prodrug of curcumin, has been successfully synthesized and investigated on its physicochemical properties and anti-inflammatory effects. CUR-2GE is characterized as a poorly soluble compound with a high partition coefficient. It is stable at acidic pH and rapidly hydrolyzes at neutral pH. The solubility of CUR-2GE can be improved using surfactants. CUR-2GE is digested in the gastrointestinal fluids and converted to CUR-1GE and curcumin readily for absorption. As a prodrug, CUR-2GE can also be bioconverted in plasma and release curcumin to exert biological activity. The potent inhibition of pro-inflammatory cytokines by CUR-2GE suggests that CUR-2GE has potential for further pre-clinical and clinical investigation on its anti-inflammatory effects for the treatment of several neurodegenerative disorders associated with neuroinflammation and microglial activation.

## Supporting information

S1 Appendix Experimental procedures. (PDF)

S1 Fig ^1^H NMR spectrum of GE-1. (TIF)

S2 Fig Mass spectrum of GE-1. (TIF)

S3 Fig ^1^H NMR spectrum of CUR-1GE. (TIF)

S4 Fig ^13^C NMR spectrum of CUR-1GE. (TIF)

S5 Fig Mass spectrum of CUR-1GE. (TIF)

S6 Fig Typical UPLC chromatograms of curcumin, CUR-1GE and CUR-2GE. The analysis of CUR-2GE, CUR-1GE, curcumin was performed on the Waters Acquity UPLC^TM^ H-Class system (Waters Corporation, MA, USA) equipped with a quaternary pump, column oven, autosampler, and photodiode array detector. The samples were separated on Acquity UPLC^TM^ BEH C18 1.7 μm, 2.1 x 50 mm column (Waters Chromatography Ireland Limited, Dublin, Ireland) at 33°C. The mobile phase consisted of 2%v/v acetic acid in water (A) and acetonitrile (B). The gradient program was used with the following profiles: initial A-B of 55:45 at 0 min; linear-gradient A-B of 20:80 from 0–2.7 min; isocratic A-B of 20:80 from 2.7–4.5 min; linear-gradient A-B of 55:45 from 4.5–5.0 min; isocratic A-B of 55:45 from 5.0–7.0 min. The flow rate was 0.3 mL/min, and the injection volume was 2 μL. The DAD detector was set at 400 nm. The Waters Empower^TM^ 3 software was used for system control and data processing. The retention times of curcumin, CUR-1GE and CUR-2GE were 1.6, 2.3 and 2.8 min, respectively.
(TIF)

S7 Fig ^1^H NMR spectrum of CUR-2GE. (TIF)

S8 Fig ^13^C NMR spectrum of CUR-2GE. (TIF)

S9 Fig Mass spectrum of CUR-2GE. (TIF)

S10 Fig Powder X-ray diffraction (PXRD) spectrum of CUR-2GE. (TIF)

S11 Fig A chromatogram of CUR-2GE in buffer pH 1.2 with 0.5% SLS for 2 h at 37°C. The UPLC method was performed on the Waters Acquity UPLC^TM^ H-Class system. The samples were separated on Acquity UPLC^TM^ BEH C18 1.7 μm, 2.1 x 50 mm column. The DAD detector was set at 400 nm. The retention times of compound 1, compound 2, CUR-1GE and CUR-2GE were 0.9, 2.0, 2.3 and 2.8 min, respectively.
(TIF)

S12 Fig Mass spectra of precursor and product ions of standards. (A) CUR-2GE, (B) CUR-1GE and (C) curcumin.
(TIF)

S1 Table Composition of CUR-2GE and its hydrolytic products as a percentage peak area in buffer pH 1.2, 4.5, and 6.8 with 0.5% SLS for 2 h at 37°C. (PDF)
