# Supplementary material for: Physicochemical investigation of a novel curcumin diethyl γ-aminobutyrate, a carbamate ester prodrug of curcumin with enhanced anti-neuroinflammatory activity
Source: PLoS One. 2022 Mar 18;17(3):e0265689. doi: 10.1371/journal.pone.0265689 (PMC9048745; doi:10.1371/journal.pone.0265689)
Supplement: S1 Appendix — (PDF) [file pone.0265689.s001.pdf]

S1 Appendix. Experimental procedures

Solubility

The solubility category was classified according to USP [1] as very soluble (>1000 mg/mL), freely soluble (100-1000 mg/mL), soluble (33-100 mg/mL), sparingly soluble (10-33 mg/mL), slightly soluble (1-10 mg/mL), very slightly soluble (0.1-1 mg/mL) and practically insoluble (<0.1 mg/mL).

The percentage of CUR-2GE in the undissociated form at various pH values were calculated based on Henderson-Hasselbalch [2] using Eq. 1:

$$\%\text{Undissociated form} = 100 - \frac{\text{antilog}\,(\text{pH-p}K_a)}{1+\text{antilog}\,(\text{pH - p}K_a)} \times 100 \qquad (1)$$

Where pH; pH value of the medium for solubilization, pK_a; negative logarithm of the dissociation constant (K_a) of CUR-2GE with computed using the Marvin (Product version: 21.8.0) of ChemAxon (Budapest, Hungary) (http://www.chemaxon.com) accessed on June 2021.

The dose number (D_0) was calculated to assign the BSC solubility class, the D_0 for each pH buffer solution without surfactant was calculated using the following Eq. 2:

$$D_0 = \frac{M_0}{C_s V_0} \qquad (2)$$

Where M_0 is the highest dose strength (mg) of capsule equivalent to curcumin, the adopted dose of CUR-2GE is 927 mg equivalent to curcumin 500 mg dose [3]. V_0 is the initial gastric volume (≈250 mL), C_s is the saturation solubility of CUR-2GE in buffer solution without surfactant (mg/mL). Drugs with $D_0 < 1$ in pH 1.2, 4.5 and 6.8 are considered as high solubility drugs. Drugs with $D_0 > 1$ in any of the buffer solutions are classified as low solubility drugs [4-10].

Partition coefficient

The $P_{o/w}$ value and $P_{o/buffer\ pH\ 4.5}$ value were calculated by Eq. 3 and Eq. 4, respectively. The Log $P_{o/w}$ and Log $P_{o/buffer\ pH\ 4.5}$ were evaluated from the average results (n = 6) from three solvent ratios, where the results fall within the range of variation of ± 0.3 units.

$$P_{o/w} = \frac{C_{n\text{-octanol}}}{C_{water}} \tag{3}$$

$$P_{o/buffer\ pH\ 4.5} = \frac{C_{n\text{-octanol}}}{C_{buffer\ pH\ 4.5}} \tag{4}$$

Where $C_{n\text{-octanol}}$ is the equilibrium concentrations of dissolved CUR-2GE in n-octanol; C_{water} and $C_{buffer\ pH\ 4.5}$ is the equilibrium concentrations of dissolved CUR-2GE in water and buffer solution pH 4.5, respectively.

Chemical stability

The linear slopes of the natural logarithm of a concentration against time were used to calculate the degradation rate (k) for the pseudo-first-order (Eq. 5).

$$\mathrm{Ln}\ [A_t] = \mathrm{Ln}\ [A_0] - kt \tag{5}$$

Where A_0 is the CUR-2GE concentration at time 0, A_t is the concentration of CUR-2GE at time t, and k is the degradation rate constant of CUR-2GE.

The half-lives ($t_{1/2}$) of CUR-2GE were calculated by Eq. 6:

$$t_{1/2} = \frac{0.693}{k} \tag{6}$$

UHPLC analysis

The standard curve for CUR-2GE in diluent (2% acetic acid: acetonitrile, 20:80) was linear over the concentration range of 0.05-12 μg/mL with r^2 >0.999 and used for determining the solubility, partition coefficient and stability of CUR-2GE.

References

1. USP. General notices and requirements. Description and Solubility. Rockville (MD): United States Pharmacopeial Convention2015.

2. Po HN, Senozan NM. The Henderson-Hasselbalch equation: Its history and limitations. Journal of chemical education. 2001;78(11):1499. doi: 10.1021/ed078p1499.

3. Salehi B, Stojanović-Radić Z, Matejić J, Sharifi-Rad M, Anil Kumar NV, Martins N, et al. The therapeutic potential of curcumin: A review of clinical trials. European journal of medicinal chemistry. 2019;163:527-45. doi: 10.1016/j.ejmech.2018.12.016 PMID: 30553144.

4. CDER/FDA. Waiver of in vivo bioavailability and bioequivalence studies for immediate-release solid oral dosage forms based on a biopharmaceutics classification system. US Department of Health and Human Services Food and Drug Administration Center for Drug Evaluation and Research (CDER). Rockville, MD2000.

5. Dahan A, Miller JM, Amidon GL. Prediction of solubility and permeability class membership: provisional BCS classification of the world's top oral drugs. The AAPS journal. 2009;11(4):740-6. doi: 10.1208/s12248-009-9144-x PMID: 19876745.

6. delMoral-Sanchez JM, Gonzalez-Alvarez I, Gonzalez-Alvarez M, Navarro A, Bermejo M. Classification of WHO essential oral medicines for children applying a provisional pediatric

biopharmaceutics classification system. Pharmaceutics. 2019;11(11):567. doi: 10.3390/ pharmaceutics11110567 PMID: 31683740.

7. Dezani AB, Pereira TM, Caffaro AM, Reis JM, Serra CHR. Equilibrium solubility versus intrinsic dissolution: characterization of lamivudine, stavudine and zidovudine for BCS classification. Brazilian journal of pharmaceutical sciences. 2013;49(4):853-63.

8. ICH. M9 guideline on biopharmaceutics classification system-based biowaivers In Proceedings of the International Conference on Harmonization (ICH) of Technical Requirements for Registration of Pharmaceuticals for Human Use. Amsterdam, Netherlands2020.

9. Kus-Slowinska M, Wrzaskowska M, Ibragimow I, Czaklosz PI, Olejnik A, Piotrowska-Kempisty H. Solubility, permeability, and dissolution rate of naftidrofuryl oxalate based on BCS criteria. Pharmaceutics. 2020;12(12):1238. doi: 10.3390/pharmaceutics12121238 PMID: 33352674.

10. Miranda C, Aceituno A, Fernández M, Mendes G, Rodríguez Y, Llauró V, et al. ICH guideline for biopharmaceutics classification system-based biowaiver (M9): toward harmonization in Latin American countries. Pharmaceutics. 2021;13(3):363. doi: 10.3390 /pharmaceutics13030363 PMID: 33801796.
