# Supplementary figures and images for: Physicochemical investigation of a novel curcumin diethyl γ-aminobutyrate, a carbamate ester prodrug of curcumin with enhanced anti-neuroinflammatory activity
Source: PLoS One. 2022 Mar 18;17(3):e0265689. doi: 10.1371/journal.pone.0265689 (PMC9048745; doi:10.1371/journal.pone.0265689)

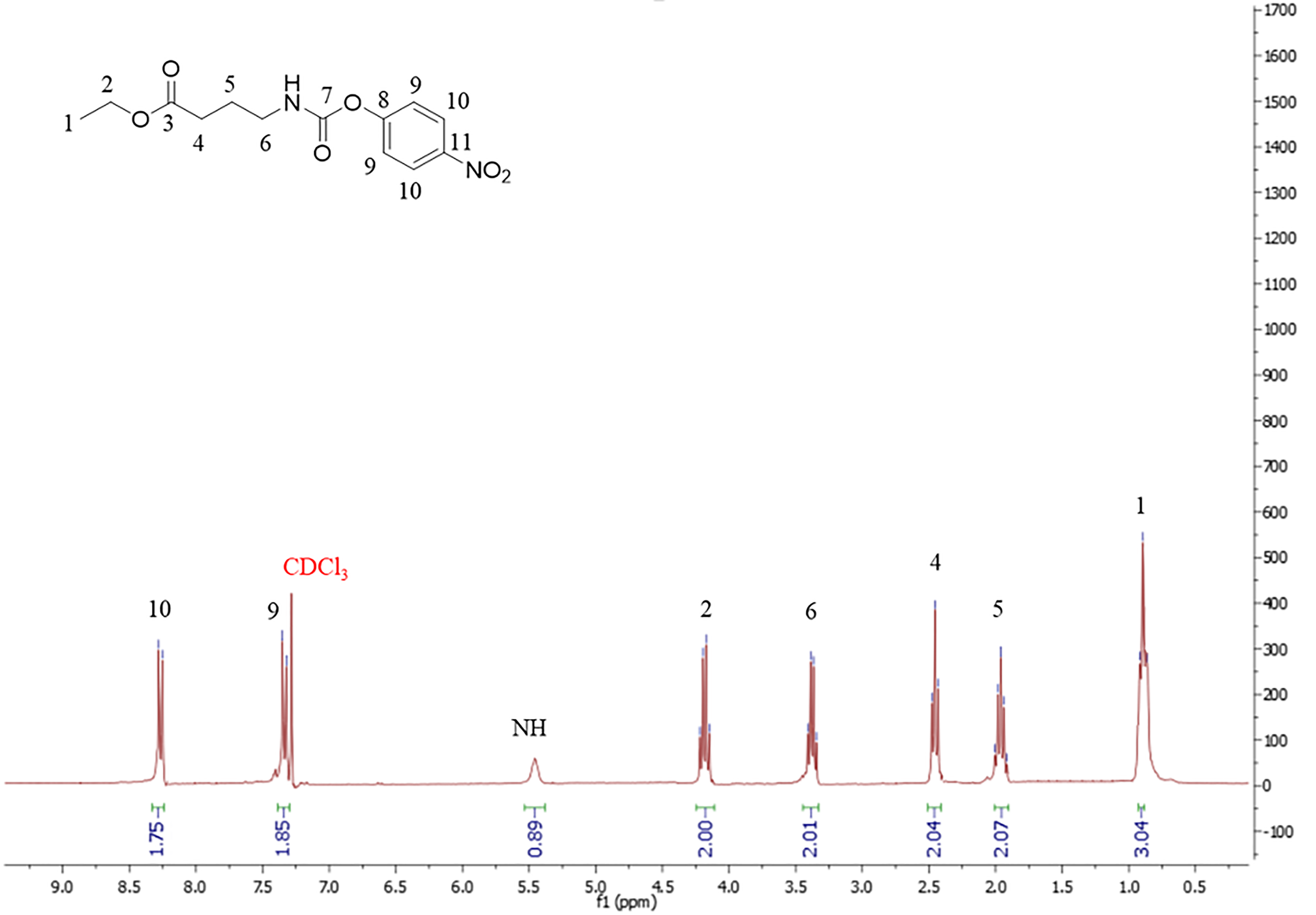

Supplement: S1 Fig — (TIF) [file pone.0265689.s002.tif]

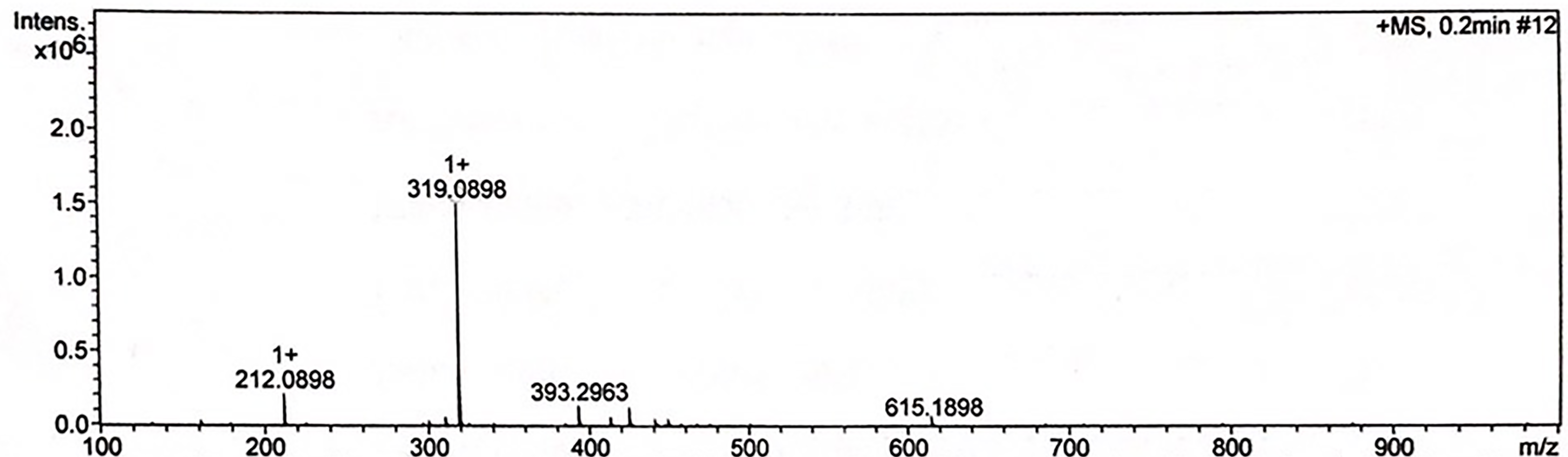

Supplement: S2 Fig — (TIF) [file pone.0265689.s003.tif]

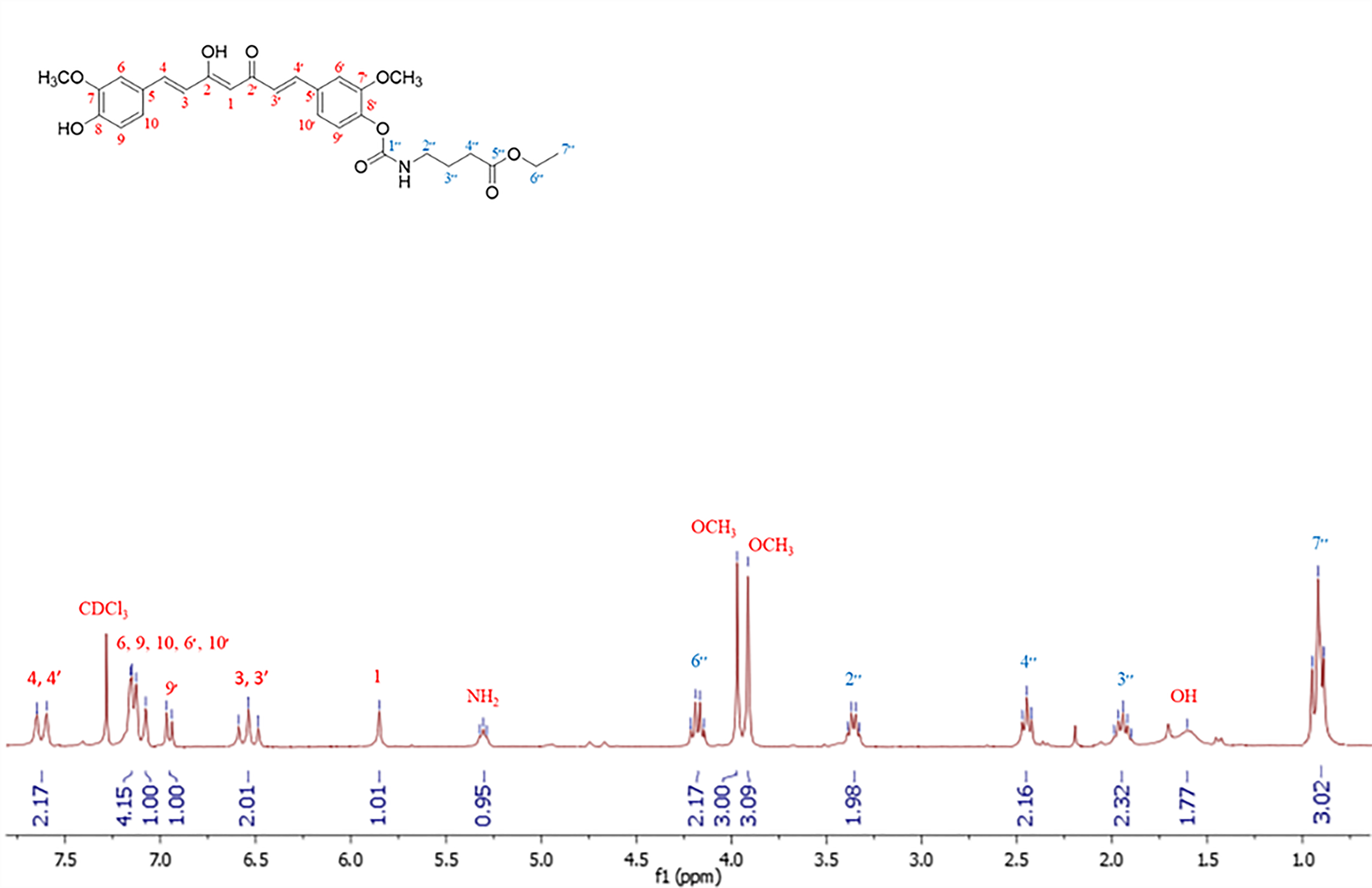

Supplement: S3 Fig — (TIF) [file pone.0265689.s004.tif]

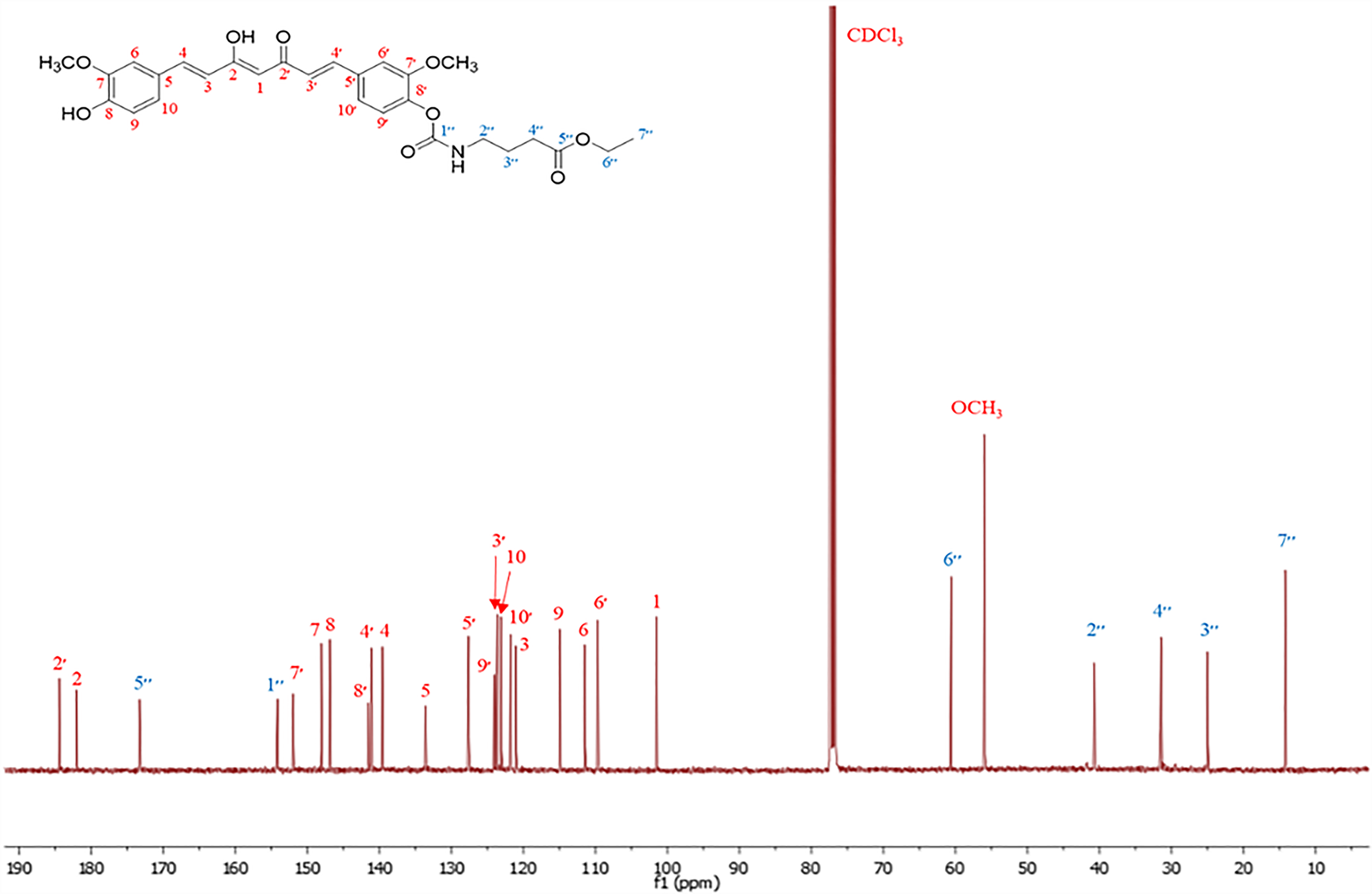

Supplement: S4 Fig — (TIF) [file pone.0265689.s005.tif]

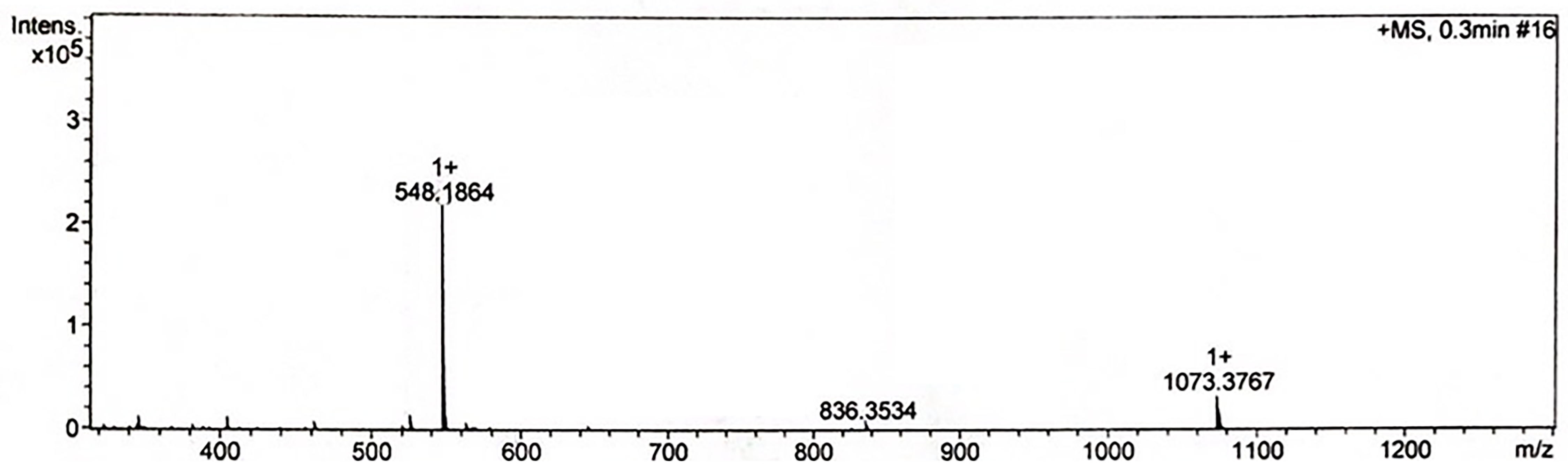

Supplement: S5 Fig — (TIF) [file pone.0265689.s006.tif]

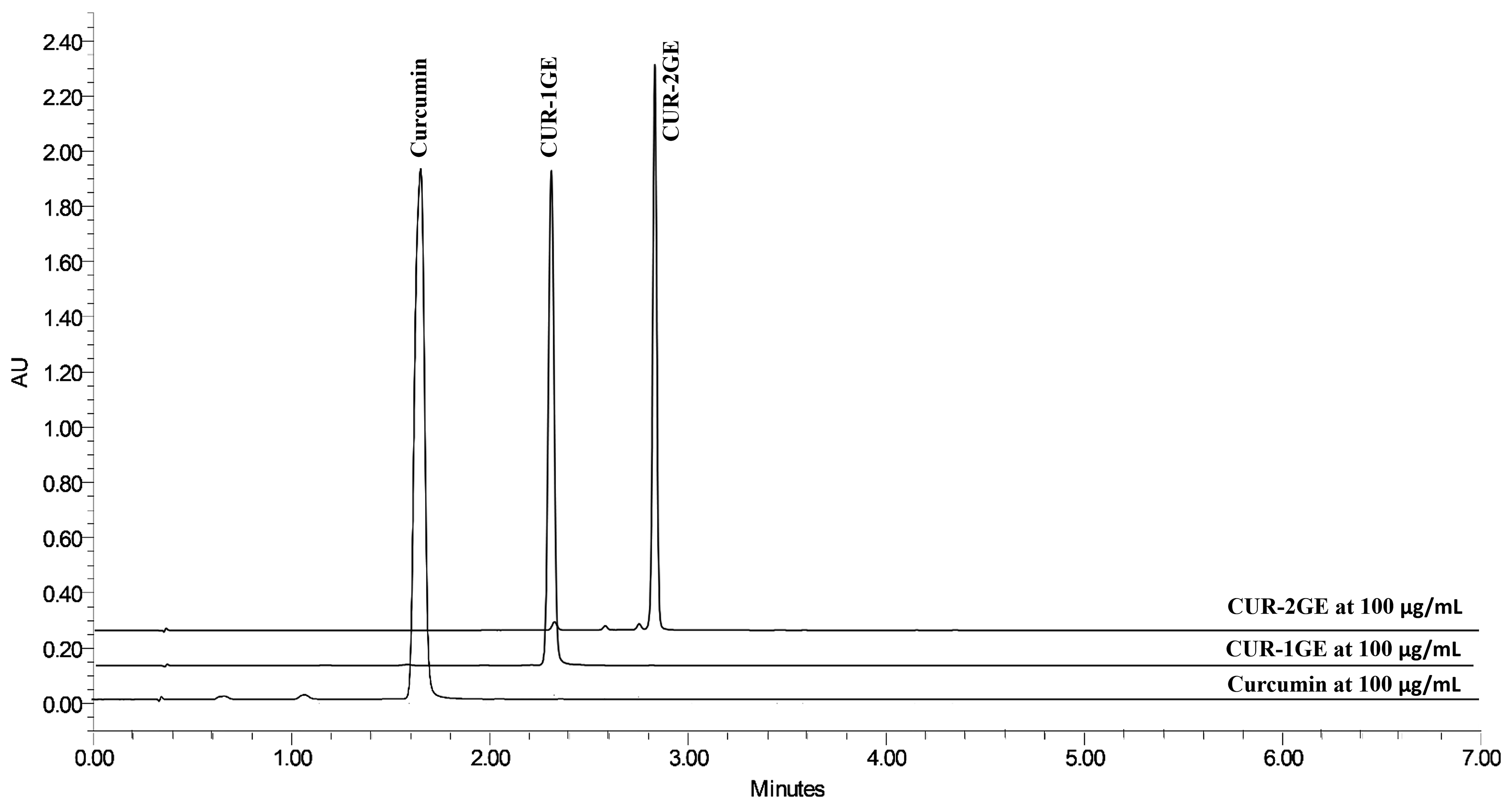

Supplement: S6 Fig — The analysis of CUR-2GE, CUR-1GE, curcumin was performed on the Waters Acquity UPLCTM H-Class system (Waters Corporation, MA, USA) equipped with a quaternary pump, column oven, autosampler, and photodiode array detector. The samples were separated on Acquity UPLCTM BEH C18 1.7 μm, 2.1 x 50 mm column (Waters Chromatography Ireland Limited, Dublin, Ireland) at 33°C. The mobile phase consisted of 2%v/v acetic acid in water (A) and acetonitrile (B). The gradient program was used with the following profiles: initial A-B of 55:45 at 0 min; linear-gradient A-B of 20:80 from 0–2.7 min; isocratic A-B of 20:80 from 2.7–4.5 min; linear-gradient A-B of 55:45 from 4.5–5.0 min; isocratic A-B of 55:45 from 5.0–7.0 min. The flow rate was 0.3 mL/min, and the injection volume was 2 μL. The DAD detector was set at 400 nm. The Waters EmpowerTM 3 software was used for system control and data processing. The retention times of curcumin, CUR-1GE and CUR-2GE were 1.6, 2.3 and 2.8 min, respectively. (TIF) [file pone.0265689.s007.tif]

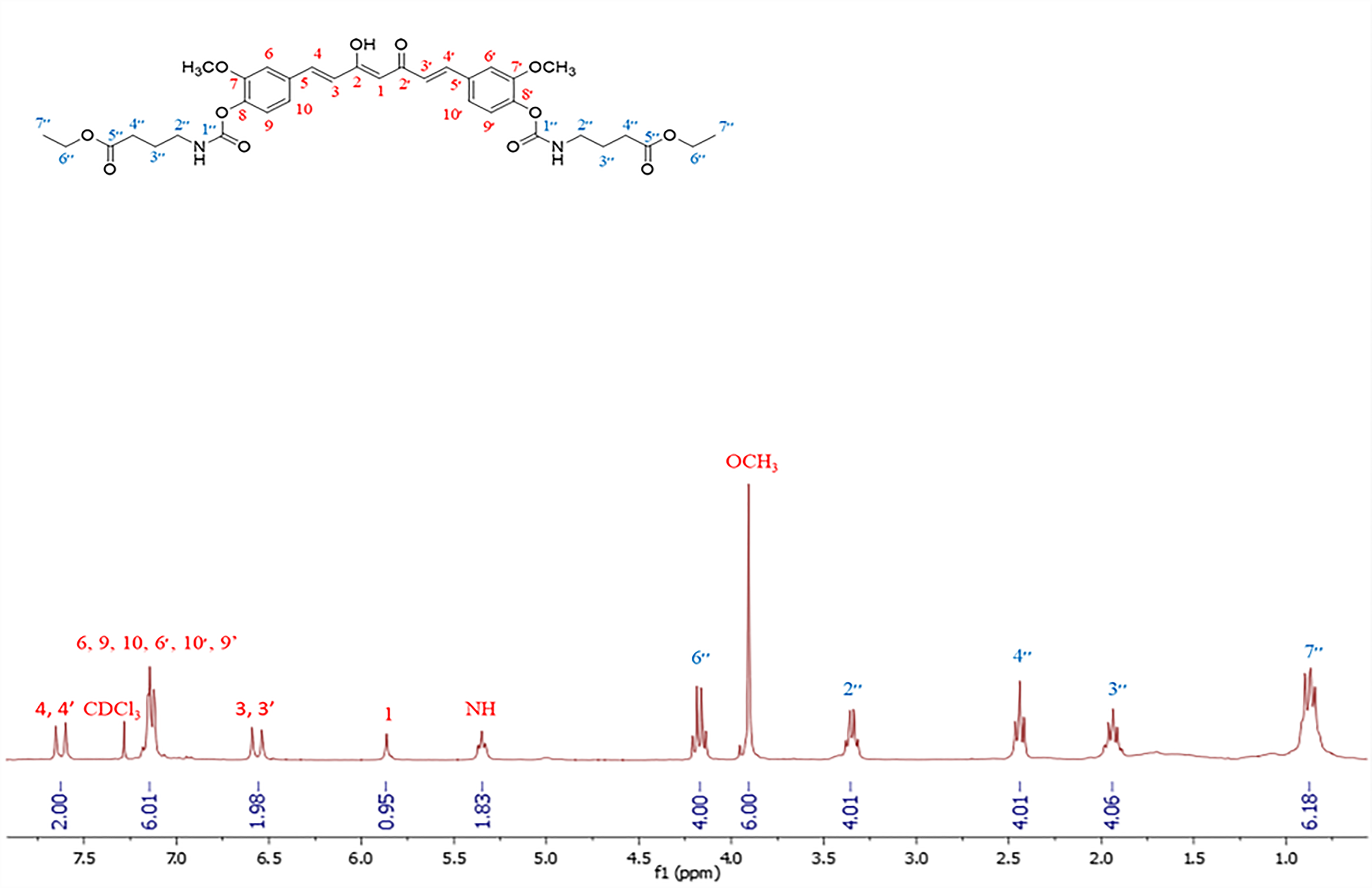

Supplement: S7 Fig — (TIF) [file pone.0265689.s008.tif]

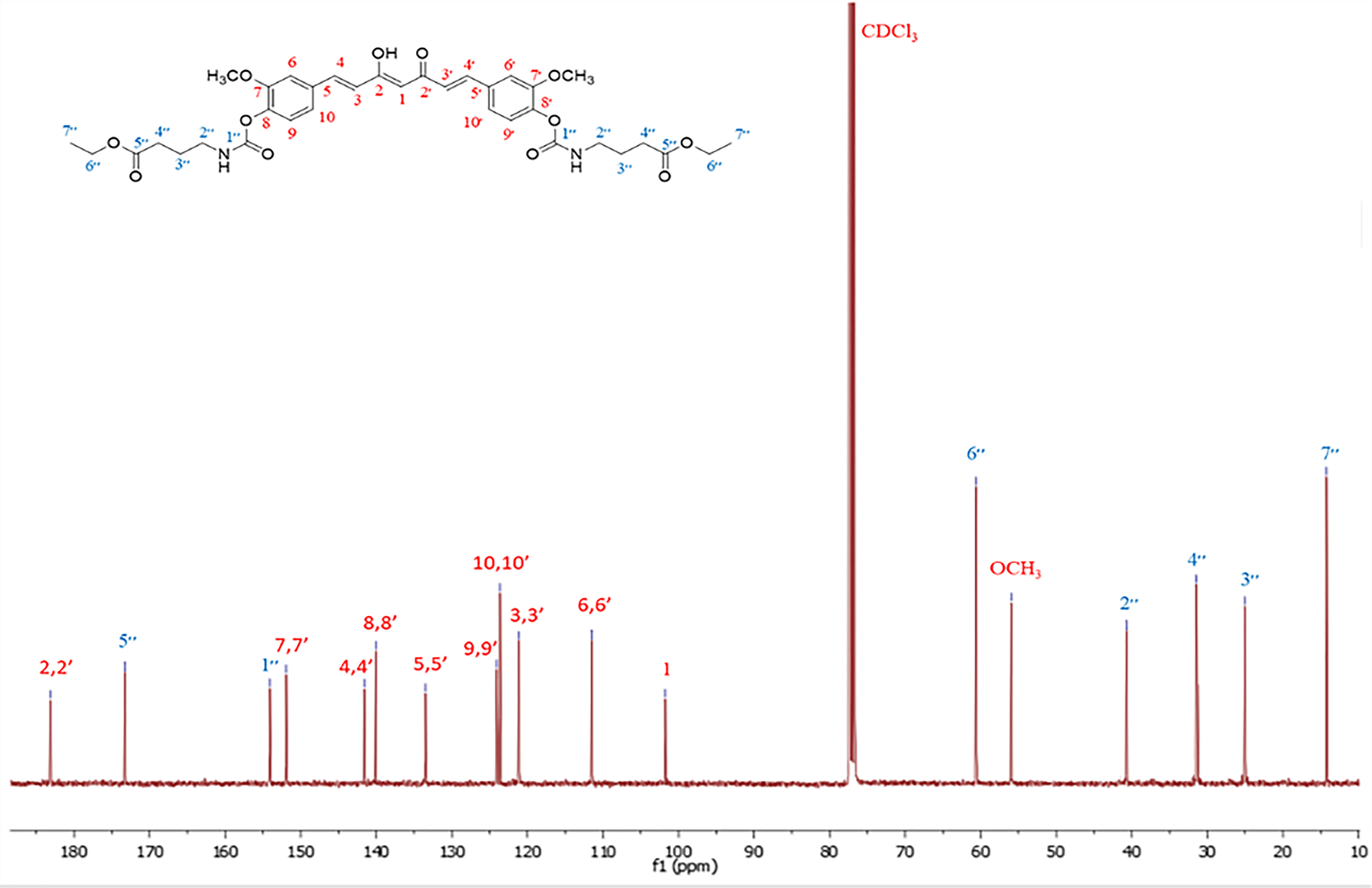

Supplement: S8 Fig — (TIF) [file pone.0265689.s009.tif]

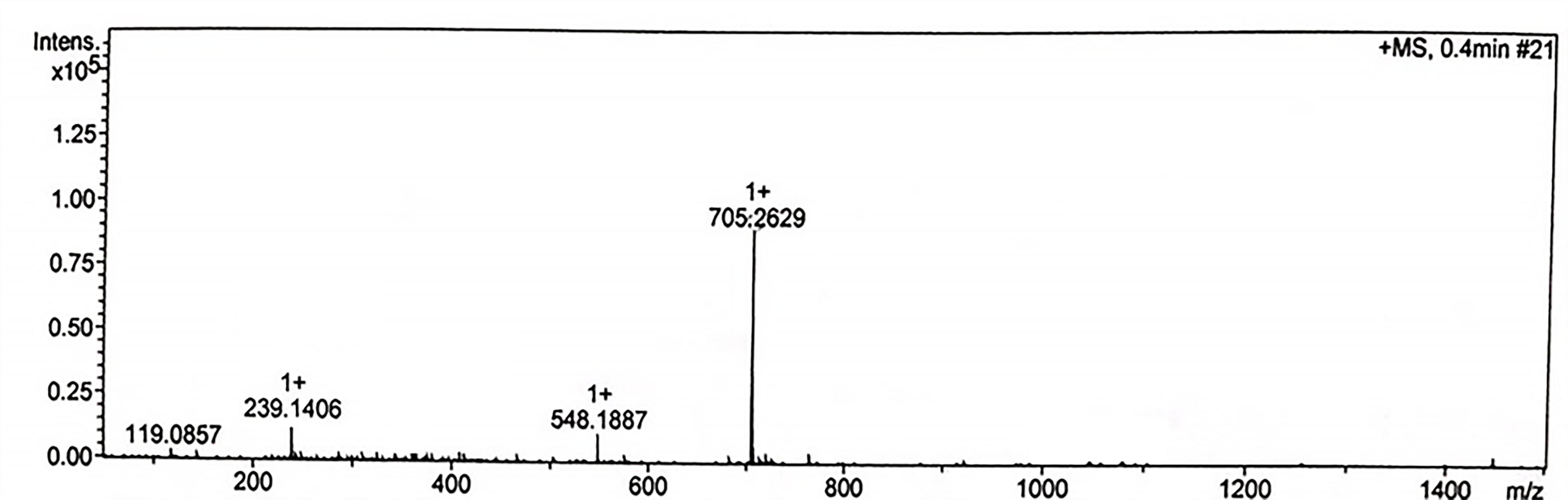

Supplement: S9 Fig — (TIF) [file pone.0265689.s010.tif]

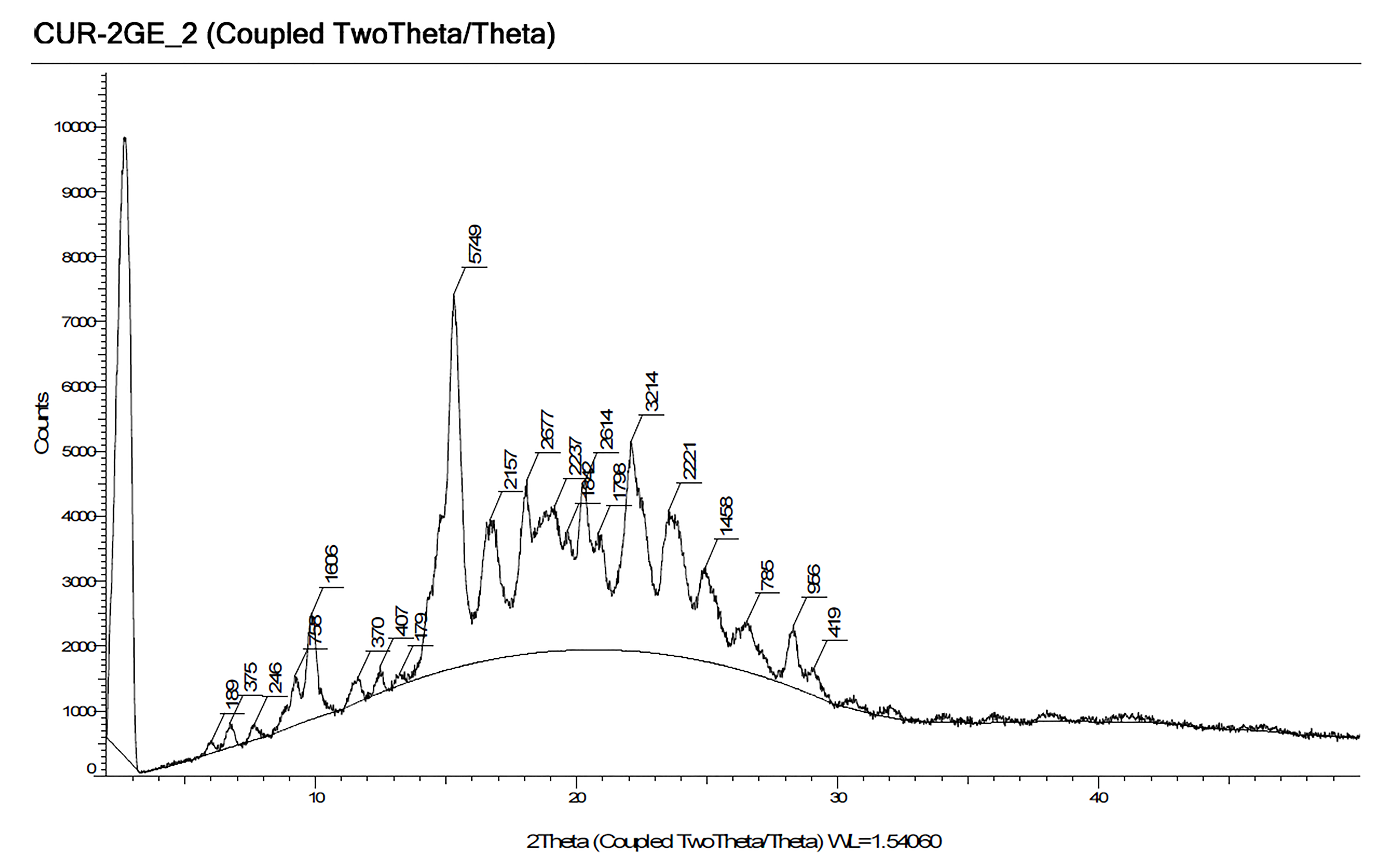

Supplement: S10 Fig — (TIF) [file pone.0265689.s011.tif]

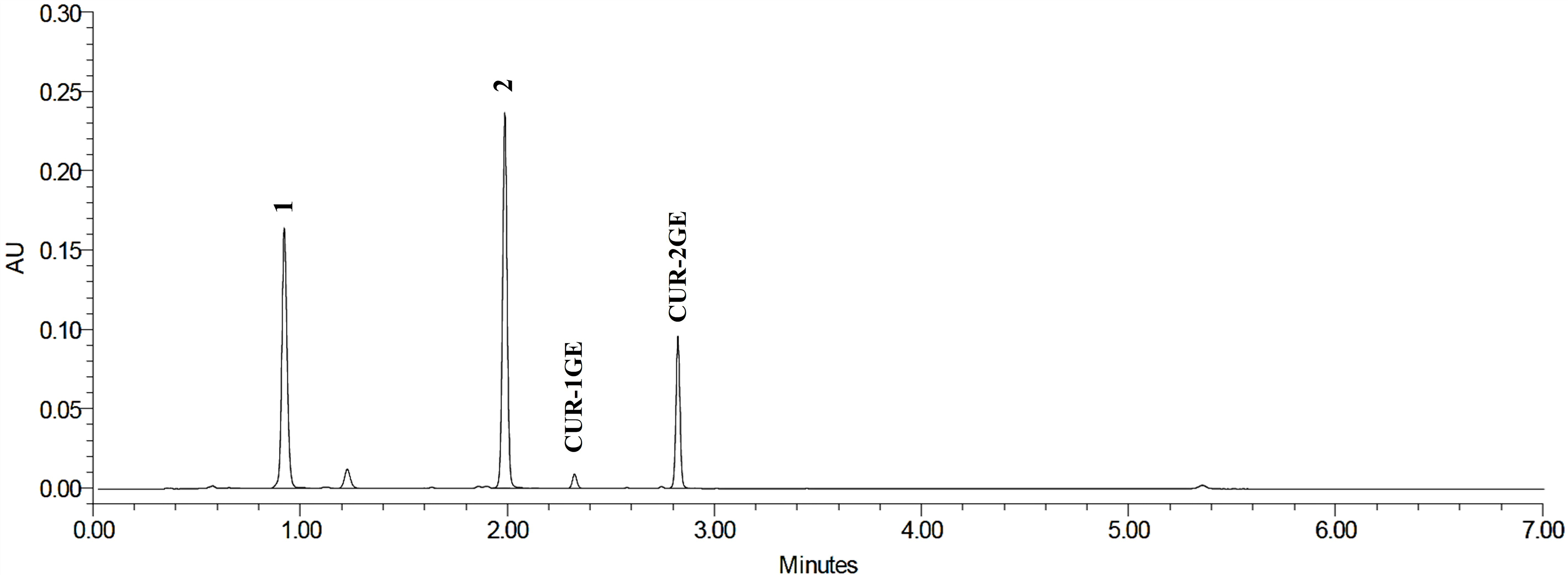

Supplement: S11 Fig — The UPLC method was performed on the Waters Acquity UPLCTM H-Class system. The samples were separated on Acquity UPLCTM BEH C18 1.7 μm, 2.1 x 50 mm column. The DAD detector was set at 400 nm. The retention times of compound 1, compound 2, CUR-1GE and CUR-2GE were 0.9, 2.0, 2.3 and 2.8 min, respectively. (TIF) [file pone.0265689.s012.tif]

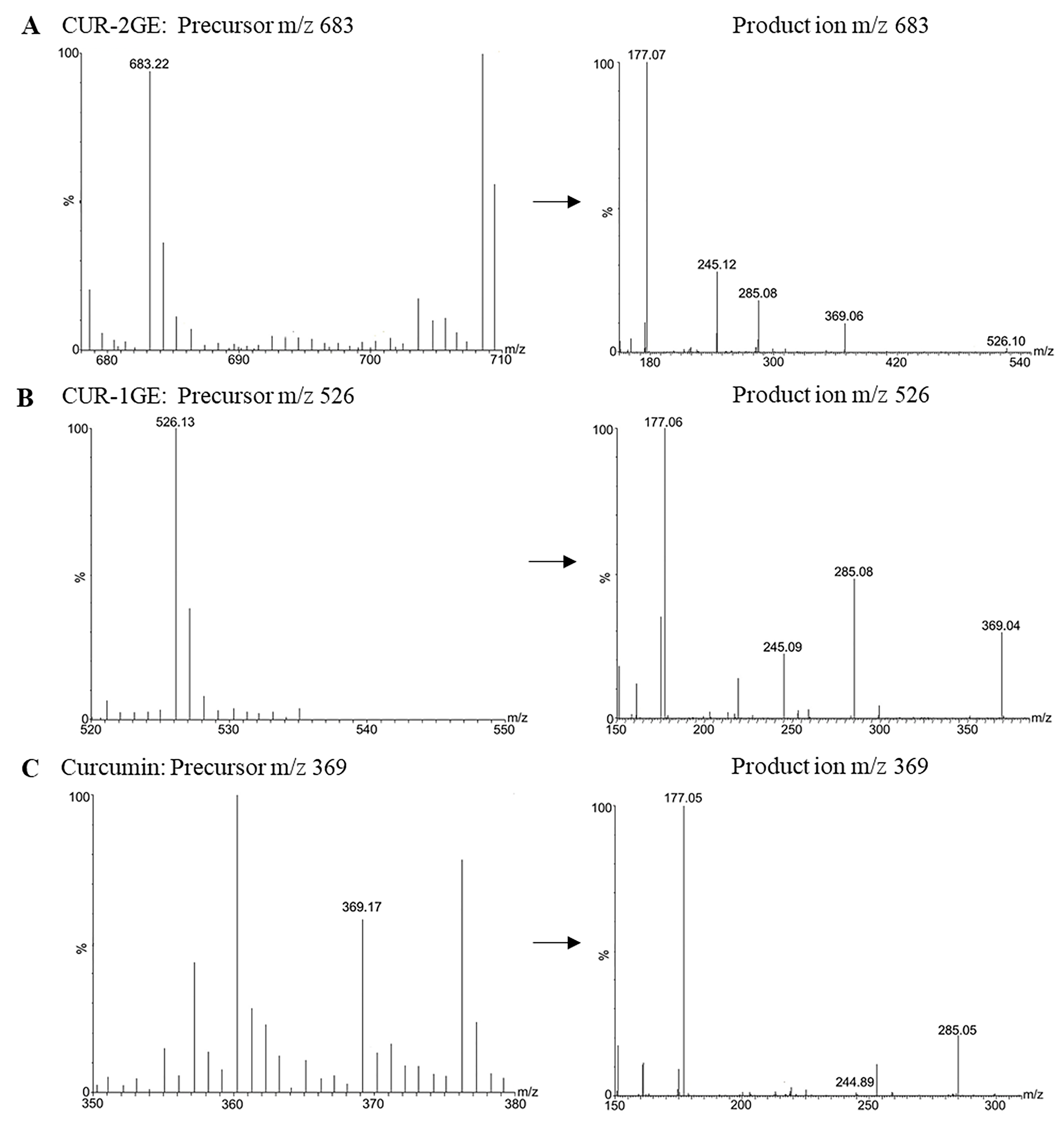

Supplement: S12 Fig — (A) CUR-2GE, (B) CUR-1GE and (C) curcumin. (TIF) [file pone.0265689.s013.tif]
