# Supplementary material for: Physicochemical investigation of a novel curcumin diethyl γ-aminobutyrate, a carbamate ester prodrug of curcumin with enhanced anti-neuroinflammatory activity
Source: PLoS One. 2022 Mar 18;17(3):e0265689. doi: 10.1371/journal.pone.0265689 (PMC9048745; doi:10.1371/journal.pone.0265689)
Supplement: S1 Table — (PDF) [file pone.0265689.s014.pdf]

S1 Table Composition of CUR-2GE and its hydrolytic products as a percentage peak area in buffer pH 1.2, 4.5, and 6.8 with 0.5% SLS for 2 hours at 37°C.

	Retention time	pH 1.2 + 0.5%SLS	pH 4.5 + 0.5% SLS	pH 6.8 + 0.5% SLS
CUR-2GE (%)	2.8	13.92 ± 1.77	93.43 ± 0.38	95.69 ± 0.07
Hydrolytic products (%)				
Compound1	0.9	37.61 ± 2.15	ND	ND
	1.2	4.37 ± 1.24	ND	ND
Compound2	2.0	41.69 ± 2.24	ND	ND
CUR-1GE	2.3	2.41 ± 0.60	6.57 ± 0.38	4.31 ± 0.07

ND = Not detected.
